# Characterization of the apoptotic response of human leukemia cells to organosulfur compounds

**DOI:** 10.1186/1471-2407-10-351

**Published:** 2010-07-02

**Authors:** W Wei-Lynn Wong, Paul C Boutros, Amanda R Wasylishen, Kristal D Guckert, Erin M O'Brien, Rebecca Griffiths, Anna R Martirosyan, Christina Bros, Igor Jurisica, Richard F Langler, Linda Z Penn

**Affiliations:** 1 Division of Cancer Genomics and Proteomics, Ontario Cancer Institute, University Health Network, Toronto, M5G 2M1, Canada; 2 Department of Medical Biophysics, University of Toronto, Toronto, M5G 2M1, Canada; 3 Division of Signaling Biology, Ontario Cancer Institute, University Health Network, Toronto, M5G 2M1, Canada; 4 Department of Computer Science, University of Toronto, Toronto, M5S 1A8, Canada; 5 Department of Chemistry, Mount Allison University, Sackville, E4L 1G8, Canada; 6 Department of Biochemistry, LaTrobe University, Australia; 7 Ontario Institute of Cancer Research, Toronto, M5G 0A3, Canada

## Abstract

**Background:**

Novel therapeutic agents that selectively induce tumor cell death are urgently needed in the clinical management of cancers. Such agents would constitute effective adjuvant approaches to traditional chemotherapy regimens. Organosulfur compounds (OSCs), such as diallyl disulfide, have demonstrated anti-proliferative effects on cancer cells. We have previously shown that synthesized relatives of dysoxysulfone, a natural OSC derived from the Fijian medicinal plant, *Dysoxylum richi*, possess tumor-specific antiproliferative effects and are thus promising lead candidates.

**Methods:**

Because our structure-activity analyses showed that regions flanking the disulfide bond mediated specificity, we synthesized 18 novel OSCs by structural modification of the most promising dysoxysulfone derivatives. These compounds were tested for anti-proliferative and apoptotic activity in both normal and leukemic cells.

**Results:**

Six OSCs exhibited tumor-specific killing, having no effect on normal bone marrow, and are thus candidates for future toxicity studies. We then employed mRNA expression profiling to characterize the mechanisms by which different OSCs induce apoptosis. Using Gene Ontology analysis we show that each OSC altered a unique set of pathways, and that these differences could be partially rationalized from a transcription factor binding site analysis. For example, five compounds altered genes with a large enrichment of p53 binding sites in their promoter regions (p < 0.0001).

**Conclusions:**

Taken together, these data establish OSCs derivatized from dysoxysulfone as a novel group of compounds for development as anti-cancer agents.

## Background

The ultimate goal of drug development is to create therapeutic agents that kill tumor cells but spare normal cells. Novel therapies that selectively induce tumor cell death are urgently needed in the clinical management of cancers. Advances in cancer biology have revealed that tumor cells retain their intrinsic ability to commit suicide, known as apoptosis [1,2]. Because of this, compounds are being developed to trigger apoptosis in a tumor-specific manner. Triggering non-inflammatory elimination of tumor cells by inducing them to undergo apoptosis is highly desirable [3].

To this end, there has been interest in exploiting the anti-proliferative effects of several natural organosulfur compounds (OSCs), such as those found in garlic, onions and mahogany trees [4,5]. Compounds from garlic, such as diallyl disulfide (DADS), have been shown to have limited toxicity and reduce tumor load *in vivo *[6]. The anti-proliferative mechanism is thought to involve cell arrest in the G2/M phase of the cell-cycle. Despite these results with natural products, chemically-synthesized OSCs have not yet been systematically evaluated for their anti-cancer activity. We have previously shown that synthesized relatives of dysoxysulfone, a natural OSC derived from the Fijian medicinal plant, *Dysoxylum richi*, also possess antiproliferative effects on tumor cells [7,8]. A structure-activity analysis of dysoxysulfone relatives identified three chemicals, termed compounds F, H and N, which did not affect the viability of normal human diploid cells but killed model leukemia cell lines [7,8]. These compounds contain a core disulfide structure similar to DADS, and our structure-activity analysis strongly suggested that regions flanking the disulfide group contribute to the specificity of cell killing. Further, these compounds are synthesized, not extracted from natural sources, providing a renewable, cost-effective and long-term manner of procuring them.

Our current study extends this previous work and attempts to determine how structure-activity relationships can be exploited to increase potency of OSCs while retaining tumor-specificity. First, we synthesized new relatives of compounds F, H and N. Next, these compounds were tested for anti-proliferative and apoptotic activity in both normal and leukemic cells. Transcriptomic profiling of potential lead compounds was then used to determine mechanism of action. Finally, these compounds were assayed for their effect on normal human bone marrow. Our results establish these synthetic OSCs as potential novel tumor-specific therapeutic agents.

## Methods

### Organosulfur Compound Synthesis

Detailed chemical synthesis and characterization are provided in Additional File 1.

### Cell Culture and Cell Lines

All cell lines, OCI-AML3 (referred to hereafter as AML-3), KK, and WI38 were assayed as asynchronously growing cells and were cultured in alpha-minimal essential medium (α-MEM) (Princess Margaret Hospital Media Services) supplemented with 10% fetal bovine serum (FBS) (Sigma, St. Louis, MO) and 1% penicillin/streptomycin at 37°C with 5% CO_2_.

### Biological Testing: General Sample Preparation

Approximately 20 mg of each compound was dissolved in ACS grade acetone (5 mL, Sigma) using a volumetric flask. Stock solutions of the compounds were stored in the dark at -20°C. Compounds were diluted, as indicated, immediately prior to each experiment.

### MTT Assays

MTT assays were completed as described previously [7]. Briefly, leukemic and WI38 fibroblast cells were seeded at 2.7 × 10^5 ^cells/mL and 0.67 × 10^5 ^cells/mL respectively in 96 well plates (Falcon, Mississauga, Ontario). The cells were exposed to an acetone control and a dose range of each compound in a total volume of 150 μL for 48 hours and assayed in triplicate. MTT data were analyzed using Prism 3.0 (GraphPad Software Inc., San Diego, CA) by the Chou-Talalay method. A dose-response curve for each compound was repeated, independently, three times.

### Fixed-PI Staining

Fixed-PI assays were completed as described previously [7]. Briefly, 10^6 ^leukemia and 2.5 × 10^5 ^WI38 cells were seeded in a 6 well dish and 30 mm plates, respectively (Falcon). Cells were then exposed for 48 hours to an acetone control and two concentrations of each compound. Cell cycle parameters were measured with Cellquest software (Becton Dickinson, San Jose, CA). Each sample was repeated, independently, three times.

### TUNEL

TUNEL assays were completed as described previously [7]. Approximately 10^6 ^leukemia cells were seeded into a 6 well dish (Falcon). The cells were then exposed to an acetone control and two concentrations of each compound for 48 hours. Cells were then harvested and fixed with 4% formaldehyde for 15 minutes on ice. Samples were analyzed using the Cellquest software (Becton Dickinson, San Jose, CA). Each sample was repeated, independently, three times.

### Colony Growth Assays

Normal bone marrow from bone marrow transplant donors was collected following informed consent according to institutional guidelines. Mono-nucleated cells were isolated from normal bone marrow using Percoll (Amersham Biosciences). Cells were washed and re-suspended in α-MEM supplemented with 10% FBS and 10% 5637 conditioned media. Conditioned media was prepared by growing the 5637 cells to confluency, replacing with fresh media and harvesting the media after three additional days of incubation. The conditioned media was used at 10% (v/v) with aMEM +10% FBS and 1% penicillin/streptomycin. Mono-nucleated cells (density: 10^6 ^cells/mL) and AML-3 cells (density: 5 × 10^5 ^cells/mL) were exposed to various concentrations of each compound for 48 hours. Cells were then washed, counted and live mononucleated (10^4^) or AML-3 (10^3^) cells were plated in Methocult (StemCell Technologies, BC, Canada). Colonies were allowed to form for 12-14 days and the number of BFU-E and CFU-C colonies of approximately >50 cells were counted. Numbers were normalized to solvent control.

### Microarray Assays

Approximately 10^7 ^AML-3 cells were treated with acetone (solvent control, 0.67% (v/v)), 180 μM H, 75 μM F, 35 μM K, 35 μM L, 100 μM N, 90 μM O, 60 μM P, 20 μM Q, 12.5 nM Taxol (Sigma) and 7.5 μM Lovastatin (Apotex). Cells were harvested for RNA at 12 hours using Trizol Reagent (Gibco Life Technologies). RNA was purified and 40 μg of RNA was used to synthesize cDNA probes and then equally aliquoted and stored at -20°C for future experiments. A home-made reference RNA set compiled of both normal and tumor cell lines (WI38, SKBR3, PANC1, SIHA, COLO320, HSR, SKMG5, ANBL-6, A549, NB-4, KK, DU145, MCF-7, WM983) was used to profile the compounds. Sample cDNA and reference cDNA were labeled using an indirect method with Cy3 and Cy5, respectively and hybridized at 42°C overnight onto an OCI 1.7 K human cDNA array. Chips were washed and then scanned using a Packard Scanner and quantified using Imagene (v5.0). Each condition was hybridized in triplicate.

### Microarray Data Analysis

The raw Imagene quantitations were read into the R statistical environment (v2.6.2) using the limma software package (v2.12.0). A series of quality assessments were performed on each array to ensure spatial and distributional homogeneity - no arrays were removed from the analysis. The array data were then normalized using the variance-stabilizing normalization (VSN) algorithm, as implemented in the vsn package for R (v3.2.1) using default parameters, with the exception of an increased iteration number of 1,000 for improved model convergence [9]. General linear modeling was then used to compare the expression profile of each drug treatment to that of the acetone control. The resulting hits were then subject to an empirical Bayes moderation of the standard error [10] and a false-discovery rate adjustment [11]. Hits were deemed differentially expressed relative to control when p_adjusted _< 0.05. Each spot on the array was re-annotated using a BLAST pipeline. Briefly, the sequence for each spot was BLASTed against the reference sequences for UniGene build Hs.199 using blastn with default parameters except for an expectation of 0.0001 and a word-size of 7, chosen to increase sensitivity of the analysis [12]. For each spot, the UniGene cluster with the highest sequence-similarity was selected as representative. UniGene clusters were then mapped to Entrez Gene IDs using annotation downloaded from NCBI on 2007-02-08. Data were clustered using divisive hierarchical algorithm DIANA with Pearson's correlation as the similarity metric, as implemented in the cluster package (v1.11.9). Heatmaps were visualized using the lattice package (v0.17-6). Thresholds for cluster analysis were selected using the F statistic from the general-linear model fit. A series of thresholds were selected to ensure that results were parameter-independent.

Gene ontology (GO) enrichment analysis employed the GOMiner software [13], using all human databases, all look-up options, all evidence codes, and all three GO ontologies. False-discovery rates were estimated using the maximum 1,000 permutations, and a threshold of 0.1 was used to select terms. A separate GO analysis was performed for each compound, and the results were merged in a database to create a matrix with GO-terms as rows and compounds as columns. This matrix was subjected to divisive hierarchical clustering using the DIANA algorithm with Pearson's correlation as the similarity metric, as implemented in the cluster package (v1.11.9). Heatmaps were visualized using the lattice package (v0.17-6). Clustering was performed on subsets of this matrix selected based on cumulative probability thresholds, where the cumulative probability was calculated as the product of the probabilities for each compound. Missing values were treated as having a probability of 1.0 at this step. A series of thresholds were selected to ensure that results were insensitive to perturbations in this parameter.

To determine if the observed changes in mRNA abundance were indicative of altered transcription factor activity, we performed transcription factor binding site (TFBS) enrichment analysis. Briefly, for each compound, we identified the set of genes exhibiting altered mRNA abundances at p_adjusted _< 0.05. For each gene we extracted 2001 bp of regulatory sequence, centered on the TFBS, using build hg18 of the human genome and the genomic positioning annotation provided by the UCSC Genome Browser Database in the REFFLAT table on 2007-04-07 [14]. We used a library of 123 TFBS motifs [15] and the CLOVER software package [16] to scan these regulatory regions and to identify TFBS motifs that were enriched or depleted. To ensure statistical robustness we employed three separate permutation controls. We used standard mono- and di-nucleotide randomization. Additionally, we extracted the regulatory regions for all genes on the microarray and used these as a background dataset. Each permutation experiment was run 10,000 times, and only those TFBSs showing enrichment or depletion at p < 0.05 (for enrichment) or p > 0.95 (for depletion) by all three tests were included in the final results.

## Results

### Synthetic Considerations

Structural details for the relatives of F, H, and N prepared and tested for this study are provided in Additional File 2 and Additional File 3, Figure S1. Unsymmetrical disulfides were constructed by condensation of an appropriate sulfenyl chloride and mercaptan pair. Sulfenyl chlorides were freshly prepared from symmetrical disulfides or from unsymmetrical methyl disulfides in accord with the detailed discussion offered earlier [17]. Most ester preparations used commercial methyl thioglycollate as the mercaptan. β-Sulfone disulfide preparations required one of the β-mercaptosulfones 1 or 2.

RSO_2_CH_2_CH_2_SH

**1 **R = *p*-CH_3_(C_6_H_4_)

**2 **CH_3_

**Figure 1:**
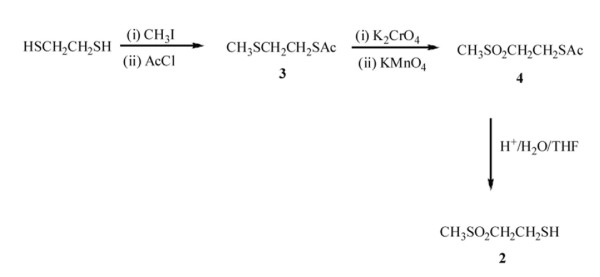
**Preparation of β-mercaptosulfone 2**. Schematic representation of the synthesis of β-mercaptosulfone 2.

The mercaptan **1 **was known [18]. The mercaptosulfone **2 **was prepared from a symmetrical starting material as outlined in Figure 1.

### Relatives of lead active OSCs reduce proliferation of leukemic cells

**Figure 2:**
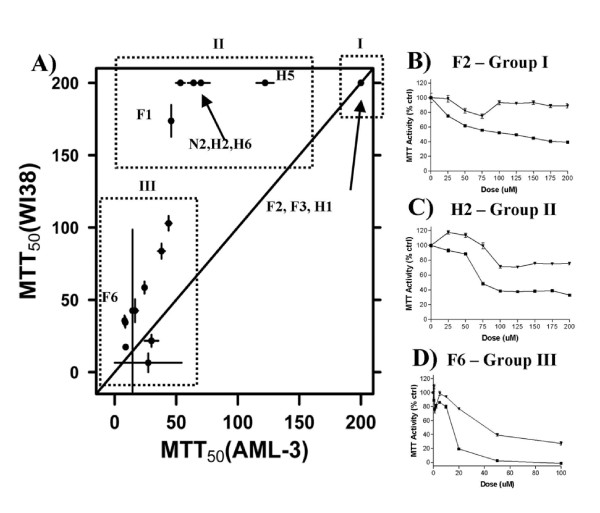
**Proliferation rates of leukemia and normal cells exposed to OSCs**. We measured the biological activity of OSCs in normal fibroblast diploid (WI38) and leukemic (AML-3) cells using the MTT assay. (A) Differential activity was observed for a subset of compounds. Group I compounds (F2, F3, and H1) are biologically inactive, Group II compounds (F1, H2, H5, H6 and N2) reduce proliferation in leukemic cells only and Group III compounds (F4, F5, F6, F7, F8, H3, H4, H7, H8 and N1) affect proliferation of both leukemic (AML-3) and normal, non-transformed cells (WI38) to varying degrees. (B-D) Representative analyses of compounds from each group: each compound was assayed in three independent experiments. AML-3 cells are shown with squares; WI38 cells are shown with triangles.

To assess the new compounds for tumor-specific anti-proliferative activity both leukemic and non-transformed human diploid fibroblast cell lines were exposed to increasing concentrations of OSCs and assayed for proliferation using the 3-(4,5-dimethylthiazol-2-yl)-2,5-diphenyltetrazolium bromide (MTT) assay [19]. The MTT assay measures the ability of cellular mitochondrial dehydrogenase to convert the yellow MTT substrate to a purple formazan product. From our previous work, the MTT_50 _values for compounds K-Q were similar across the panel of cell lines. Accordingly, we selected a representative leukemic cell line, AML-3, for subsequent MTT assays and used a non-transformed diploid cell line of fibroblast origin, WI38, as a control. A comparison of the MTT_50 _values between normal (WI38) and leukemic (AML-3) cells show that many compounds induce differential responses (Additional File 4 and Figure 2A), with enhanced activity in leukemic cells compared to normal cells being most common (values above the y = x line in Figure 2A). The MTT assays segregated the compounds into three clear and distinct groups (Figure 2A). Group I compounds (F2, F3 and H1) possessed little to no anti-proliferative activity at the concentrations tested (Figure 2B). Group II compounds (F1, H2, H5, H6, and N2) decreased proliferation of AML-3 but not of WI38 cells (Figure 2C). Based on our previous analyses, compounds F, H, K, L, N, O, P and Q are also classified in group II [8] The chemical composition of these previously evaluated compounds is provided in Table 1 for comparison. Group III compounds (F4, F5, F6, F7, F8, H3, H4, H7, H8, and N1) decreased proliferation of both AML-3 and WI38 cells to varying degrees (Figure 2D). Thus, the MTT assay indicates compounds in group II have anti-proliferative activity specific to leukemic cell lines, while compounds in group III have anti-proliferative activity that affects both normal cells and tumor cells, but to different degrees. This MTT study shows that relatives of F, H and N are more potent than the parental structure.

**Table 1:** Colony growth of leukemia cells and normal bone marrow in response to treatment with organosulfur compounds


**Group**	**Compound Name**	**Structure**	**Dose (μM)**	**Selectivity**	

				**AML**	**NBM**

I	F2	CH_3_SO_2_CH_2_CH_2_SSCH_2_CH_2_SO_2_CH_3_	ND		

	F3	CH_3_SO_2_CH_2_CH_2_SSPh	ND		

	H1	CH_3_OC(O)CH_2_CH_2_SSCH_3_	ND		

II	F	CH_3_SO_2_CH_2_CH_2_SSCH_3_	100	++	+

	F1	CH_3_SO_2_CH_2_CH_2_SSCH_2_CO_2_CH_3_	100	+++	+

	**H**	**CH**_**3**_**OC(O)CH**_**2**_**SSCH**_**3**_	**175**	**++**	-

	H2	CH_3_OC(O)CH_2_SSCH_2_CH_2_CH_3_	50	++	+

	**H5**	**CH**_**3**_**OC(O)CH**_**2**_**SS(CH**_**2**_**)**_**6**_**CH**_**3**_	**200**	**+++**	-

	H6	CH_3_(CH_2_)_5_OC(O)CH_2_SSCH_2_CH_2_CH_3_	50	+	+

	K	CH_3_SO_2_CH_2_SSCH_2_CH_2_CH_2_CH_2_CH_3_	50	+++	++

	**L**	**CH**_**3**_**SO**_**2**_**SPh**	**50**	**++**	-

	**N**	**CH**_**3**_**SCH**_**2**_**SSCH**_**3**_	**100**	**++**	-

	N2	CH_3_SCH_2_SSCH_2_CH_2_CH_2_SO_2_CH_3_	50	++	+

	**O**	**CH**_**3**_**OC(O)CH**_**2**_**SSCH**_**2**_**C(O)OCH**_**3**_	**100**	**++**	-

	**P**	**PhC(O)CH**_**2**_**SSCH**_**3**_	**100**	**++**	-

	Q	PhSSCH_2_OC(O)CH_2_CH_3_	50	++	++

III	F4	CH_3_SO_2_CH_2_CH_2_SS(C_6_H_4_)OCH_3_	20	++	+

	F5	CH_3_SO_2_CH_2_CH_2_SS(C_6_H_4_)NO_2_	20	+++	+++

	F6	CH_3_(C_6_H_4_)SO_2_CH_2_CH_2_SSCH_3_	40	+++	+++

	F7	CH_3_(C_6_H_4_)SO_2_CH_2_CH_2_SSPh	30	++	+

	F8	CH_3_(C_6_H_4_)SO_2_CH_2_CH_2_SSCH_2_CH_2_SO_2_(C_6_H_4_)CH_3_	20	+++	++

	H3	CH_3_OC(O)CH_2_SS(C_6_H_4_)OCH_3_	ND		

	H4	CH_3_OC(O)CH_2_SSPh	25	+	-

	H7	CH_3_(CH_2_)_5_OC(O)CH_2_S SPh	25	+	-

	H8	CH_3_OC(O)CH_2_SS(C_6_H_4_)NO_2_	ND		

	M	O_2_N(C_6_H_4_)SSCH_3_	50	+++	+++

	N1	CH_3_SCH_2_SSCH_2_SCH_2_SO_2_CH_3_	50	+++	+++

Results of compounds assayed for specificity and sensitivity to leukemic cell lines and normal bone marrow using colony growth assay. The colony growth assay results for compounds K, L, M, N, O, P and Q were repeated a minimum of three independent times with consistent results. Colony growth assay results for the remaining compounds were repeated twice. Data is summarized as the fraction of colonies formed compared to solvent controls, and indicated as >0.75 (-, no effect), 0.75 (+), 0.50 (++), and >0.25 (+++). Compounds with tumor-specific activity are shaded. Abbreviations: AML, Acute Myelogenous Leukemia; NBM, normal bone marrow; ND, not determined

### OSCs induce changes in cell cycle and apoptosis

**Figure 3:**
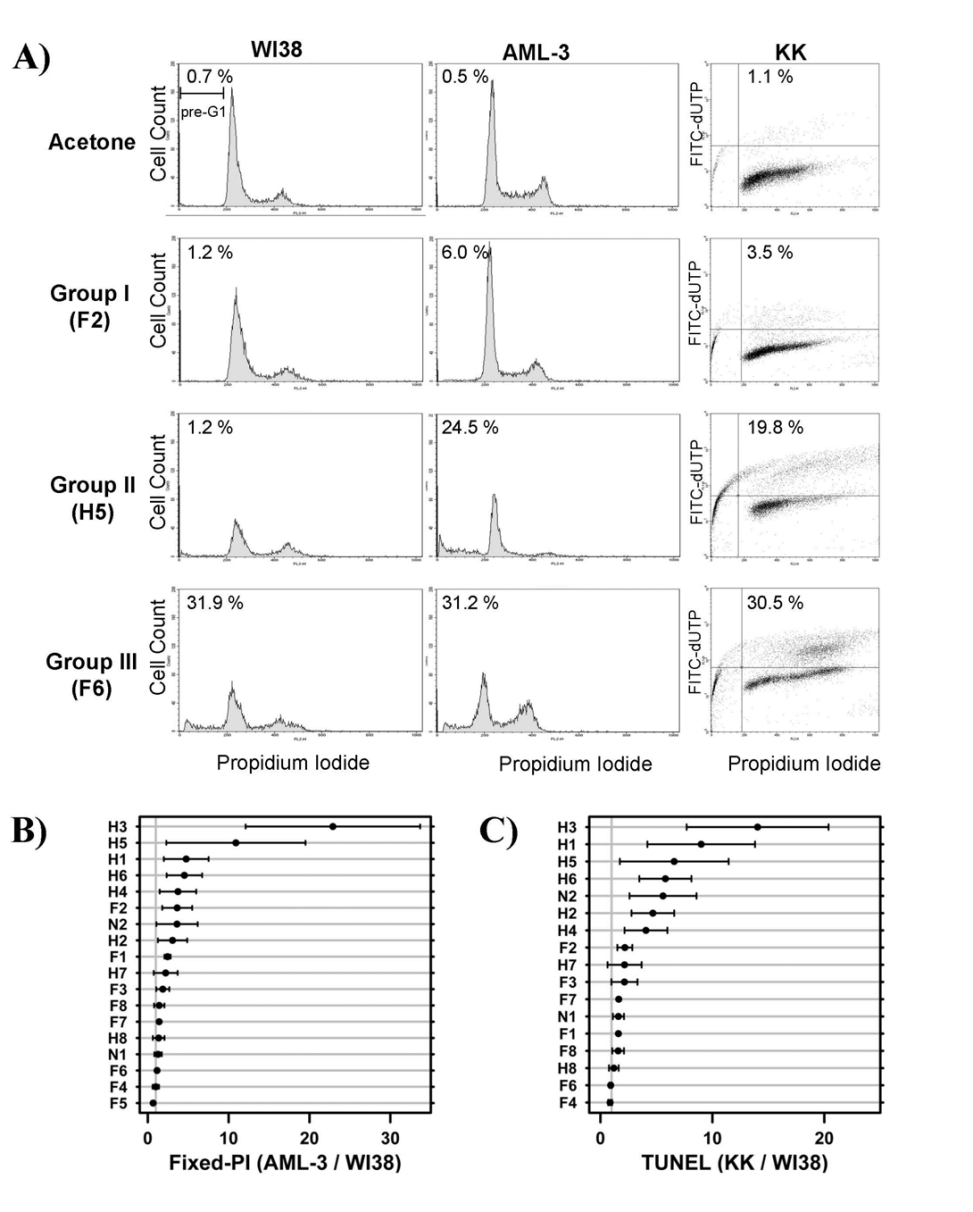
**The effects of OSCs on cell cycle profile and apoptosis**. A) The first two columns show representative cell-cycle profiles in normal WI38 and leukemic AML-3 cells after 48 hours of exposure to OSCs from each group (F2 at 200 μM, H5 at 200 μM, F6 at 80 μM). Cells were stained with propidium iodide and analyzed by flow cytometry. The percentage of cells staining in the pre-G1 region (apoptotic) is displayed in the upper left corner of each histogram. The third column shows TUNEL staining of leukemic KK cells after 48 hours of exposure to OSCs (F2 200 μM, H5 200 μM, F6 40 μM). The percentage of cells staining TUNEL positive (apoptotic) is displayed in the top right quadrant. B) The mean percentage of cells staining in pre-G1 region (apoptotic) was determined for AML-3 and WI38 cells. The ratio of these two values for each OSC is a proxy for their therapeutic ratio. A similar analysis was performed for KK cells (Additional File 1 Figure S2A). C) The mean percentage of cells staining TUNEL positive (apoptotic) was determined for each OSC in both KK and WI38 cells. The ratio of these two values is a proxy for their therapeutic ratio. A similar analysis was performed for AML-3 cells (Additional File 1 Figure S2B).

To determine whether the observed anti-proliferative activity was a consequence of elevated apoptosis, both leukemic (AML-3 and KK) and non-transformed fibroblast (WI38) cells were exposed to OSCs at two concentrations for 48 hours. We evaluated the proportion of apoptotic cells in leukemic and normal cell lines by fixed-PI (Figure 3B and Additional File 3, Figure S2A) and terminal deoxynucleotidyl transferase-mediated dUTP\nicked-end-labeling (TUNEL) (Figure 3C and Additional File 3, Figure S2B) assays. The combined results of these experiments are provided in Additional File 5. As expected, compounds from group I were biologically inactive and did not affect apoptosis in any cell line, as assayed by both fixed-PI and TUNEL (Additional File 5 and Figure 3), confirming the MTT assay. Group II compounds, with the exception of F1, preferentially induced apoptosis in leukemic cells over normal diploid cells (Additional File 5 and Figure 3). Compound F1 induced apoptosis in a concentration dependent manner in both leukemic and normal diploid cells, despite the difference in anti-proliferation shown by the MTT assay. TUNEL experiments independently confirmed the leukemic cells were undergoing apoptosis from each phase of the cycle upon exposure to compounds F1, H2, H5 and H6 (Additional File 5, Figure 3).

Interestingly, as demonstrated previously for compounds K, L, N, O, P and Q, some OSCs (H2, H5 and H6) changed the cell cycle profile of the WI38 cells, with an accumulation of cells in the G_2_/M phase of the cell cycle, suggesting that OSCs can trigger G_2 _cell cycle arrest in non-transformed cells (Figure 3A and data not shown).

Compounds from group III could be classified into two distinct groups based on their cell cycle profiles in leukemic and normal cells (Figure 3C). Compounds H3, H7, and N1 increased the percentage of cells in the pre-G1 region for both leukemic and normal diploid cells but were more toxic to leukemic cells (Additional File 5). These compounds caused leukemic cells to stain TUNEL positive in all phases of the cell cycle. In direct contrast, at the concentrations tested, the remaining compounds (F4, F5, F6, F7, F8, H4 and H8) similarly induced apoptosis in both the leukemic cell lines and the normal diploid cells (Additional File 5 and Figure 3). Apoptosis was confirmed in the leukemic cell lines by TUNEL staining at the concentrations used and counterstained with PI to examine cell cycle profile (Additional File 5). Of the compounds tested in group III, only compound F6 caused an increase in the number of cells in the G2/M phase of the cell cycle (Figure 3).

Based on the combined results from the MTT, fixed-PI and TUNEL assays we grouped OSCs into the following categories. Group I, inactive compounds are F2, F3 and H1. Group II, tumor-selective agents are compounds F1, H2, H5, H6, N2 as well as compounds F, H, K, L, N, O, P and Q from our previous work [7,8]. Group III compounds, which do not exhibit tumor-specific activity like compound M from our previous study, are compounds F4, F5, F6, F7, F8, H3, H4, H7, H8, and N1.

### Expression profiling segregates OSCs into mechanistic sub-classes

We performed cDNA microarray analysis to evaluate the mechanisms by which OSCs induce tumor-specific apoptosis. AML-3 cells were exposed to compounds from group II - F (75 μM), H (180 μM), L (35 μM), K (35 μM), N (100 μM), O (90 μM), P (60 μM), and Q (20 μM). Concentrations were selected that induce approximately 20-30% apoptosis in 48 hours as determined by the TUNEL assay (data not shown). To capture the molecular events occurring prior to apoptosis, cells were harvested after 12 hours of exposure and RNA was extracted. As positive controls for apoptosis, two other compounds were analyzed: lovastatin and taxol. Lovastatin is a well-known treatment for hypercholesterolemia and has been identified as a tumor-specific inducer of apoptosis [20]. Taxol is a chemotherapeutic agent that induces apoptosis in the G2/M phase of the cell cycle [21].

We used a rigorous quality-control assessment of each array (Additional File 3, Figure S3) and pre-processed the data using standard techniques. For each compound, general linear-modeling was used to identify specific genes whose mRNA abundances were significantly altered (p_adjusted _< 0.05) relative to the acetone control; complete results are presented in Additional File 6.

**Figure 4:**
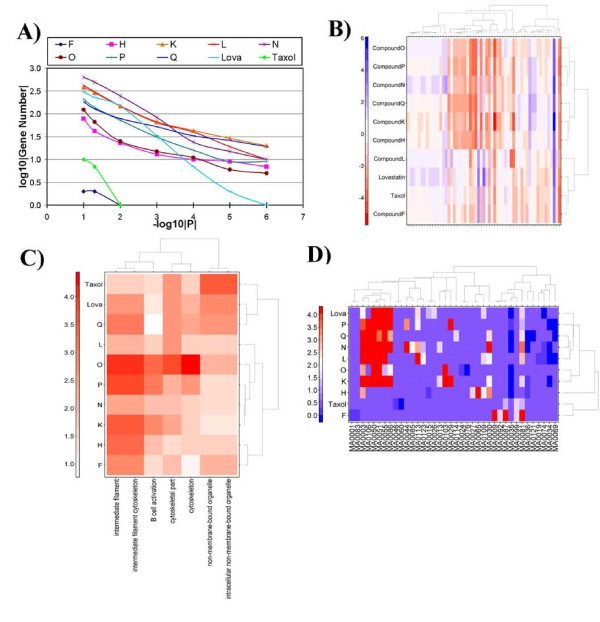
**Microarray profiling of OSCs identifies mechanistic groups**. (A) Microarray analysis of changes in mRNA abundance induced by 8 OSCs and two control compounds identifies that some compounds induce significantly greater alterations than others. (B) These profiles of changes in mRNA abundances can be used to clusters the compounds using a divisive hierarchical algorithm (colour bars represent fold-change relative to acetone control). (C) Mapping altered mRNA profiles to functional groups allows clustering of compounds based on their functional profiles (colour bars represent -log_10_|P|). (D) To assess the mechanistic underpinnings of altered mRNA abundance profiles we performed transcription factor binding site analysis, and clustered compounds into mechanistic groups (colour bars represent -log_10_|P|, labels indicate difference TFBS IDs). Intriguingly, the mRNA abundance, GO functional, and TFBS mechanistic results allow clustering of the data into similar, but non-identical groups.

The number of genes affected by each compound varies across several orders of magnitude (Figure 4A). At the concentrations and time-points selected both compound F and taxol significantly alter the mRNA levels of tens of genes whereas compounds N and K alter hundreds.

Compounds with similar mRNA profiles might share mechanisms of action. Differentially expressed genes (p_unadjusted _< 10^-12^; F-test) were subjected to unsupervised pattern recognition (Figure 4B) [22]. Three groups of compounds exist as defined by the ratio of within-cluster to between-cluster distances: one containing compounds N, O, and P; one containing compounds H, K, and Q; and one containing compound F along with lovastatin and taxol; compound L is an outlier. We verified the parameter-independence [23] of our analysis by varying the p-value threshold from 10^-3 ^to 10^-12 ^(Additional File 3, Figures S4-S6).

Knowing that the OSCs induced compound-specific changes in mRNA abundance profiles, we sought to determine if these changes could be related to specific functional pathways using gene ontology (GO) enrichment analysis. This involves determining if the genes whose mRNA abundances are significantly altered (p_adjusted _< 0.05) are enriched for specific functional annotations relative to the array as a whole. A well-established, publicly available algorithm and implementation were used for this analysis [13]. For each compound we identified a set of functional annotations that were enriched. Selected enriched GO terms are given in Table 2 (the complete list is available as Additional File 7).

**Table 2:** Selected top enriched GO terms

ID	Term	F	H	K	L	N	O	P	Q	Lova	Taxol
GO:0003723	RNA Binding	0.0	1.2	3.0	0.3	1.9	0.4	0.1	7.2	0.2	0.0

GO:0006412	Translation	0.0	1.2	4.4	1.1	5.1	0.4	0.7	5.8	1.2	0.0

GO:0045765	Regulation of angiogenesis	0.0	0.0	1.1	1.1	1.1	3.7	0.2	1.4	0.3	0.0

GO:0030162	Regulation of proteolysis	0.0	0.0	1.2	0.4	0.3	0.0	0.0	0.7	3.6	0.0

GO:0043122	Regulation of I-kappaB kinase NF-kappaB cascade	0.0	0.0	0.6	3.3	0.4	0.0	0.4	0.0	1.3	0.0

GO:0046870	Cadmium ion binding	0.0	0.0	0.0	0.5	1.3	3.1	0.0	0.8	0.0	2.3

GO:0006935	Chemotaxis	1.8	0.5	2.0	0.4	1.5	0.9	1.5	0.7	2.9	1.3

Genes that were differentially expressed (p_adjusted _< 0.05) by each compound were subjected to Gene Ontology enrichment analysis using the GOMiner tool with 1,000 permutations. The -log_10_|P| for selected GO terms are shown here. For example, genes annotated as being involved in RNA binding are enriched amongst those differentially expressed by compound Q with p < 10^-7.2 ^and amongst those differentially expressed by compound H with p < 10^-1.2^.

Having determined that the compounds could be grouped according to their mRNA abundance profiles, we hypothesized that these groups could be recapitulated from their pathway profiles. We used the log_10_|P| values from the GO enrichment analysis for terms showing enrichment at P_cumulative _< 10^-10 ^to group compounds using divisive hierarchical clustering (Figure 4C). Pathway-based clustering yielded nearly-identical patterns of mRNA-profile-based clustering (compare Figures 4B and 4C), with the exception of compound N. To verify this finding statistically we calculated the Rand and Adjusted Rand indices. These metrics give a direct assessment of similarity between the two clustering profiles, with values of one indicating identical clusters and values of zero indicating random-chance similarity. The Rand-Index between mRNA- and GO-derived groupings was 0.89 while the Adjusted Rand-Index was 0.67, verifying the similarity. We again ensured the stability of our clustering patterns by varying the threshold used to select GO terms across several orders of magnitude (Additional File 3, Figures S7-S9).

Our microarray data identified both specific genes and specific functions altered by each OSC. We hypothesized that alterations in mRNA abundances (and hence alterations in functional profiles) are regulated, in part, by specific transcription factors. To test this, we analyzed transcription factor binding site (TFBS) enrichment [24]. This analysis identified 38 specific DNA sequence motifs that are enriched in genes responsive to one or more compounds (Additional File 8). The average motif was enriched in 3 ± 2 compounds, indicative of very different transcriptional regulatory networks.

Some patterns of TFBS enrichment are suggestive of mechanisms of apoptosis. For example the p53 binding site was enriched amongst genes dysregulated by five compounds (K, L, N, P, and Q). Given its powerful pro-apoptotic role, these data might indicate p53-mediated apoptosis. Similarly, the target genes of compound O were depleted for binding sites of the growth-promoting E2F family of transcription factors.

To determine if patterns of transcriptional regulation could be used to group compounds we subjected the set of enriched TFBSs to divisive hierarchical clustering (Figure 4D). Interestingly, while GO analysis led to a similar organization of compounds compared to mRNA data alone (Figure 4 parts B and C), TFBS analysis did not. The adjusted Rand-index between the TFBS clustering and the mRNA based clustering was 0.0, and between the TFBS clustering and the GO clustering was only 0.1 - dramatically lower than the 0.7 concordance between mRNA and GO clusters. Although TFBS is a relatively limited technique for probing transcriptional regulatory networks, these data suggests that different compounds perturb a common set of biological pathways, but by different regulatory mechanisms.

### Six OSCs preserve normal myeloid progenitor colony formation

To determine the potential of progenitor cells to repopulate the hematopoietic system after exposure to OSCs we employed colony growth assays. Normal bone marrow was exposed to various doses of OSCs for 48 hours, washed, and counted. Simultaneously, an equivalent number of cells were plated in methylcellulose supplemented with growth factors, without any OSC exposure. After 12-14 days colonies of myeloid progenitors were counted, normalized to the solvent control and compared to the colony forming potential of similarly treated AML-3 cells. Due to a limited availability of normal bone marrow, group I (inactive) compounds were not assayed. Two concentrations were used when possible to determine if the compound was truly selective for leukemic cells.

Of the group II compounds (compounds F, F1, H, H2, H5, H6, K, L, N, N2, O, P and Q), seven (F, F1, H2, H6, K, N2 and Q) reduced the proliferative potential of normal myeloid progenitors (Table 1) and thus have evidence for normal bone marrow toxicity. Importantly, six OSCs (compounds H, H5, L, N, O and P) did not markedly reduce the proliferative potential of normal myeloid progenitors, providing further evidence of their tumor specificity.

Group III compounds (F4, F5, F6, F7, F8, H4, H7, and N1) were used at lower concentrations than in previous assays to confirm our findings that these compounds were acting as general cytotoxins. Colony growth assays of group III compounds showed equivalent reduction of colony forming potential in leukemic and normal bone marrow, corroborating our previous findings (Table 1).

## Discussion

Our previous work identified organosulfur compounds derived from a Fijian medicinal plant, *Dysoxylum richi*, as potential tumor-specific anti-proliferative agents. A structure-activity analysis identified groups adjacent to the disulfide bond as being important mediators of tumor-specificity. Accordingly, we set out to design and synthesize novel relatives of the most effective previously tested compounds. We then tested those compounds for anti-proliferative and apoptosis-inducing ability in both normal (albeit of fibroblast rather than hematopoietic origin) and tumor cells. Finally, we performed microarray analysis to map the pathways regulated by each compound.

To characterize these novel organosulfur compounds we first assayed for anti-proliferative activity using the MTT assay over a wide range of concentrations. These assays were performed in both normal and tumor cells to ensure tumor-specificity. We then tested compounds for apoptotic potential using two distinct approaches: fixed-PI and TUNEL. These assays allowed assessment of the effects of each OSC on cell cycle progression. Finally, for selected compounds we used colony growth assays with normal human bone marrow to verify tumor-specificity in a more physiological model system.

Combining the results from these different assays, the 18 novel compounds fall into three groups. First, three compounds were biologically inactive (F2, F3, and H1). Second, five compounds showed some tumor-specific induction of apoptosis (F1, H2, H5 H6, and N2),. Finally, the remaining ten compounds (F4, F5, F6, F7, F8, H3, H4, H7, H8 and N1) induced apoptosis with little or no selectivity between tumor and normal cells. It should be noted that these findings are potentially dose-dependent: biologically inactive compounds may show activity at higher concentrations than employed here.

Apoptosis induced by some disulfide-containing compounds, such as ajoene, diallyl disulfide or dithiosulfinates, are under investigation. Some studies have been associated with the induction of reactive oxygen species, inhibition of metabolic enzymes, DNA modification and cell signal disruption [4,25,26]. The mechanism by which the novel OSCs induce apoptosis is currently unknown, and so we performed a microarray study to address this issue. Our microarray analyses assessed eight OSCs and two control substances, taxol and lovastatin, at a single dose and with triplicate measurements. Our analysis of this dataset found great variability in the number of genes whose mRNA levels were altered by each compound. Some compounds, like F and Taxol, only altered the expression of a few genes at the doses used; other compounds, like N and K, altered the expression of hundreds of genes.

To test if the mRNA changes induced by different compounds represent distinct functional groups we employed a gene-ontology functional analysis. Clustering of functional profiles (Figure 4C) led to a similar hierarchical organization to that found by clustering the raw expression profiles (Figure 4B). Several key biological processes were identified as dysregulated by this analysis. For example, compounds K, N, and Q all perturbed the expression of a large number of genes involved in translation. By contrast, lovastatin dysregulated genes involved in proteolysis and compound L dysregulated genes annotated as being positive regulators of the I-kappa-B kinase NF-kappa-B cascade. These results provide insight into the specific intra-cellular mechanisms of each compound.

The fact that specific functional groups were perturbed by each compound suggested that the compounds might be altering the activity of specific transcription factors, either directly or indirectly through feedback loops. To test this hypothesis we employed TFBS enrichment analysis [24] using a database of 130 known TFBS motifs. Several binding site motifs were enriched in a compound-specific manner, including strong enrichment of the PPAR-gamma motif in genes regulated by compound H. Intriguingly, genes dysregulated by compounds K, L, N, P and Q were enriched for p53 binding sites, suggesting these compounds may induce p53-dependent apoptosis.

**Figure 5:**
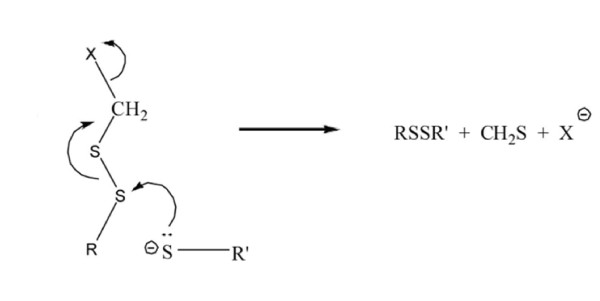
**Structure-activity model for the synthesis of novel organosulfur compounds**.

Combined, we synthesized 18 novel organosulfur compounds, beginning with CO-linked α-ester and α-sulfone disulfides [27]. A good structure-activity correlation was observed for the assumption that these compounds behave as activated electrophiles (see Figure 5) that react selectively with biochemically-significant thiol moieties.

**Figure 6:**
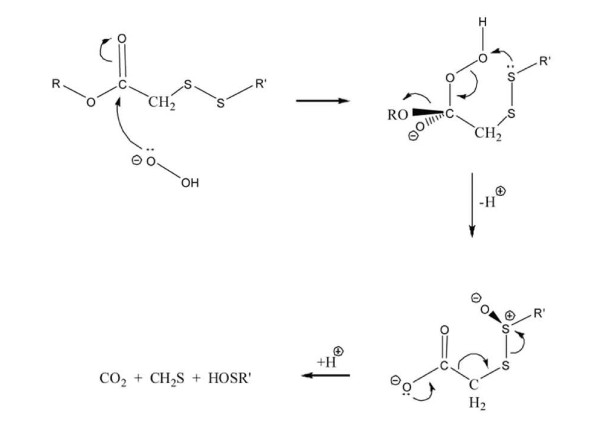
**Proposed chemical mechanisms for the activity of organosulfur compounds**.

The successful application of the rationale presented in Figure 5 not only led to the design of esters and sulfones having substantially enhanced antifungal activity [28] but also permitted the extension of our bioactive organosulfur compounds to include two new classes i.e. aryl methyl disulfides (e.g. M) and structurally related thiosulfonates (e.g. L). Subsequent work established that selected disulfides showed promise as agents with antileukemic activity [7,8], antimalarial activity [29], antithrombotic activity [30,31] and inhibitory activity against phagocytosis of anti-Rh(D)-coated red blood cells [32,33]. Despite its utility in our previous OSC studies, our new data for compounds F1 → F8 and H1 → H8 suggest that the rationale in Figure 5 is mechanistically incomplete. To that end, the following points are salient. (i) All of the compounds with tumor-specific activity (F1, H2, H5, H6) are CC-linked α-ester disulfides. (ii) The compounds with the highest therapeutic indices have longer alkyl substituents (i.e. H5). (iii) The CC-linked β-ester disulfide (H1) is inactive. (iv) Aggressive SS activation in accord with the Figure 5 view leads to a loss of selectivity (i.e. H8 vs. H). Longer alkyl substituents (C_3 _→ C_7_) enhance lipophilicity for the α-ester disulfides which enhances intracellular transport. Somewhat diminished activity for H6 (compared to H5), when the alkyl group is moved from sulfenyl sulfur to ester oxygen, hinders approach to the carbonyl carbon, suggesting that it may be serving as an electrophile. A proposed chemical mechanism that accounts for the foregoing points is presented in Figure 6.

Both thioformaldehyde and sulfenic acids are well-known traps for mercaptide anions and would act to sequester biochemically significant SH groups. Note that the proposed internal attack of a disulfide sulfenyl sulfur on the peroxide linkage (step 2, Figure 6) goes through a favorable 6-membered transition state. Application of the Figure 6 mechanism to the β-ester disulfide H1 would require the corresponding internal attack to proceed through an unfavorable 7-membered transition state, thus accounting for the inactivity of H1. Moreover, the proposed function of the carbonyl group in coordinating a peroxy anion leads one to expect superior efficacy for the α-ketodisulfide, P in accordance with our microarray observations. Overall, we believe this proposed mechanism of action accounts for our observed structure-activity results. Moreover, it serves as a platform from which future synthetic targets can be modelled. In future studies it will also be valuable to evaluate how other structural factors, such as lipophilicity, alter the biological activity of OSCs. Our previous structure-activity studies identified several promising OSCs (F, H, K, L, N, O, P and Q). These molecules were all classified as tumor-selective group II compounds.

## Conclusions

In this manuscript we have characterized the anti-proliferative effects of 18 newly synthesized OSCs, and identified an additional five group II compounds (F1, H2, H5, H6, N2). Combined, we believe H, H5, L, N, O and P show the most promise. These six OSCs were able to induce apoptosis and reduce colony formation of leukemic cells. Importantly, they had little effect on colony formation of normal myeloid progenitor cells, suggesting that they have a therapeutic window of activity. Moving forward, we believe these compounds are suitable for preliminary toxicology testing. These studies will be an essential component to further advance these OSCs as potential anti-cancer therapeutics. Additionally, an improved understanding of the pharmacokinetics of these compounds may allow determination of the potential therapeutic index required for tumor specificity *in vivo*. While our results indicate that micro-molar concentrations would be required to achieve apoptosis in leukemic but not normal cells, the maximum achievable plasma concentration of each compound has yet to be determined. It is possible that a slightly less active compound will have a higher achievable dose, making it more suitable for further study. An alternative approach would be sustained low-dose delivery, but the efficacy of this approach for organosulfur compounds remains untested. While compounds F, F1, H3, H7, K, N2, and Q are not suitable for animal testing, the information gleaned by comparing their activity to their structure will be important for the development of new compounds. A new round of structure-activity analysis based on these data may lead to novel compounds with even higher tumor-specific activity. It is interesting to note that the compounds to move forward are not necessarily from one structural class, and it will be fascinating to determine exactly how and why the flanking regions alter potency and specificity to such an extent.

In addition to yielding promising drug candidates, this study also provides a model for academic drug discovery and design. By integrating separate synthetic chemistry and molecular biology laboratories remarkable progress was achieved towards a novel therapeutic class.

## Competing interests

The authors declare that they have no competing interests.

## Authors' contributions

WWW, RFL, and LZP conceived of and initiated the project. WWW, RG, ARM, and CB functionally characterized novel OSCs. PCB performed all bioinformatic and statistical analyses. ARW, KDG, and EMO synthesized novel OSCs. LZP, RFL, and IJ provided project guidance and supervision. WWW, PCB, and ARW wrote the first draft of the paper, which all authors edited and approved.

## Pre-publication history

The pre-publication history for this paper can be accessed here:

http://www.biomedcentral.com/1471-2407/10/351/prepub

## Supplementary Material

Additional file 1 **Supplementary Materials and Methods**. Additional descriptions of methodologies, particularly those involving chemical syntheses. Click here for file

Additional file 2 **Figures S1-S9**. Microsoft PowerPoint file containing Supplementary Figures 1-9, along with FigureLegends. Click here for file

Additional file 3 **Table S1**. List of all organosulfur compounds discussed in the manuscript. Click here for file

Additional file 4 **Table S2**. Complete MTT_50 _data. Click here for file

Additional file 5 **Table S3**. Complete Fixed-PI and TUNEL data. Click here for file

Additional file 6 **Table S4**. Complete annotated gene-list from the microarray study. Click here for file

Additional file 7 **Table S5**. Complete Gene Ontology results from the microarray study. Click here for file

Additional file 8 **Table S6**. Complete TFBS analysis from the microarray study. Click here for file
