# Supplementary material for: Characterization of the apoptotic response of human leukemia cells to organosulfur compounds
Source: BMC Cancer. 2010 Jul 2;10:351. doi: 10.1186/1471-2407-10-351 (PMC2928001; doi:10.1186/1471-2407-10-351)
Supplement: Additional file 1 — Supplementary Materials and Methods. Additional descriptions of methodologies, particularly those involving chemical syntheses. [file 1471-2407-10-351-S1.DOC]

# Supplementary Materials & Methods For: Characterization of the Apoptotic Response of Human Leukemia Cells to Organosulfur Compounds

### Wong WW^1,2,6,*^, Boutros PC^1,2,7,*^, Wasylishen AR^1,2,*^, Guckert KD^5^, O’Brien EM^5^, Griffiths R^1^, Martirosyan AR^1^, Bros C^1^, Jurisica I^2,3,4^, Langler RF^5^, Penn LZ^1,2^

^1^ Division of Cancer Genomics and Proteomics, Ontario Cancer Institute, University Health Network, Toronto, Canada, M5G 2M1

^2^ Department of Medical Biophysics, University of Toronto, Toronto, Canada, M5G 2M1

^3^ Division of Signaling Biology, Ontario Cancer Institute, University Health Network, Toronto, Canada, M5G 2M1

^4^ Department of Computer Science, University of Toronto, Toronto, Canada, M5S 1A8

^5^ Department of Chemistry, Mount Allison University, Sackville, Canada, E4L 1G8

^6^ Current Address: Department of Biochemistry, LaTrobe University, Australia

^7^ Current Address: Ontario Institute of Cancer Research, Toronto, Canada

^*^ These authors contributed equally to the work

Requests for reprints to:

Dr. Linda Z. Penn

Ontario Cancer Institute

Room 9-625

610 University Avenue

Toronto, Ontario

M5G 2M9

Phone number: (416) 946-2276

Fax Number: (416) 946-2840

Email: lpenn@uhnres.utoronto.ca

Instrumental and routine procedural details for the spectroscopic characterization of our samples have been provided previously (1).

*Preparation of mercaptosulfone* **1**

Details for the preparation and characterization of **1** have been provided earlier (2).

*Multistep preparation of mercaptosulfone* **2**

Part A. Sodium metal (1.22 g, 53.0 mmol) was dissolved in methanol (100 mL) and 1,2-ethanedithiol (4.96 g, 53.0 mmol) was added. The reaction mixture was cooled with an ice-water bath. A solution of methyl iodide (6.97 g, 50.0 mmol) in methanol (20 mL) was added drop-wise. The reaction mixture was stirred at ambient temperature overnight. Hydrochloric acid (10%, 5 mL) and water (120 mL) were added and the aqueous layer extracted with methylene chloride (three 75 mL aliquots). The combined organic layers were washed with sodium hydroxide (2.5% W/V, three - 50 mL aliquots). The combined aqueous extracts were acidified with concentrated hydrochloric acid and extracted with methylene chloride (three - 75 mL portions). These combined organic layers were dried (MgSO_4_), filtered and concentrated to furnish a mixture (2.99 g) of unchanged 1,2-ethanedithiol and CH_3_SCH_2_CH_2_SH.

Part B. The mercaptan mixture from Part A was added to acetyl chloride (30 mL) and the reaction mixture refluxed for 30 min. The solvent was evaporated and the residue chromatographed on silica gel (200 g) employing 1:1 chloroform/petroleum ether (100 mL fractions) for elution. Fractions 5-7 were concentrated and combined and the residue rectified at reduced pressure affording thiolacetate sulfide **3** (2.00 g, 13.3 mmol, 25%, bp 62-68°C/100 Torr). The thiolacetate sulfide **3** had IR 1682 cm^-1^; ^1^H NMR (270 MHz) δ 2.17 (3H, s), 2.35 (3H, s), 2.66 (2H, t), 3.09 (2H, t); ^13^C NMR δ 15.3, 28.6, 30.6, 33.8, 195.1; GCMS 150 (M^+.^, 43%), 74 (100%), 61 (44%), 43 (49%).

Part C. Sulfide thiolacetate **3** (4.00 g, 26.6 mmol) and potassium chromate (5.51 g, 28.3 mmol) were added to glacial acetic acid (600 mL) and the reaction refluxed for 15 minutes. The reaction flask was immersed in an ice/water bath until acetic acid crystals began to form. Chloroform (2 L) was added and the resultant mixture extracted with 10% W/V sodium hydroxide (four 1L aliquots). The organic layer was dried (MgSO_4_), filtered and the solvent evaporated. Crude product was dissolved in acetone (320 mL) and the solution cooled in an ice-water bath. Anhydrous magnesium sulfate (28.4 g) and acetone (80 mL) were added to the reaction mixture. At ambient temperature, potassium permanganate (3.74 g) was added in three portions over 1.5 h. The reaction mixture was filtered through a celite pad. Sodium thiosulfate (100 g) was added and the reaction mixture stirred at ambient temperature for 20 minutes. The reaction mixture was filtered and then treated with thiosulfate four more times which removed most of the colour due to unreacted permanganate. The solvent was evaporated and the residue dissolved in chloroform (150 mL), dried (MgSO_4_), filtered and concentrated. The pale yellow residue was rectified at reduced pressure giving the sulfone thiolacetate **4** (1.44 g, 8.1 mmol, 30%, bp 167-170°C/2.5 Torr, mp 55-57°C). The sulfone thiolacetate **4** had IR 1685, 1305, 1133 cm^-1^; ^1^H NMR (60 MHz) δ 2.40 (3H, s), 3.06 (3H, s), 3.33 (4H, broad s); GCMS 182 (M^+.^, 0.4%), 123 (36%), 103 (17%), 43 (100%).

Part D. Sulfone thiolacetate **4** (0.51 g, 2.7 mmol) was dissolved in tetrahydrofuran (40 mL) and water (10 mL) was added. Concentrated sulfuric acid (0.5 mL) was added and the reaction mixture refluxed for 24 h. Chloroform (100 mL) was added and the resultant mixture extracted with 2.5% W/V sodium hydroxide (three 100 mL portions). The aqueous layers were combined and concentrated hydrochloric acid was added until the solution was strongly acidified. The combined aqueous layers were extracted with chloroform (three 75 mL portions).The organic layers were combined, dried (MgSO_4_), filtered and the solvent evaporated. Mercaptosulfone **2** was rectified at reduced pressure affording clean **2** (0.16 g, 1.12 mmol, 41%, bp 148-152°C/2.2 Torr). The mercaptosulfone **2** had IR 2566, 1309, 1124 cm^-1^; ^1^H NMR (270 MHz) δ 1.87 (1H, t, J=8.1 Hz, SH), 2.96 (2H, m, CH_2_), 2.99 (3H, s), 3.34 (2H, t, J=8.1 Hz, CH_2_); ^13^C NMR δ 17.1, 41.4, 57.9; GCMS 140 (M^+.^, 1.5%), 81 (100%), 61 (80%).

*Preparation of the precursor for F4*

The precursor for F4, *p*-methoxyphenyl methyl disulfide, was prepared as follows. Sodium metal (0.16 g, 6.9 mmol) was dissolved in methanol (20 mL) and *p*-methoxythiophenol (1.0 g, 7.1 mmol) was added. The solvent was evaporated and the residue dried *in vacuo*. The dried salt was dissolved in dimethyl sulfoxide (3 mL) and dimethyl disulfide (7 mL) was added. The reaction mixture was stirred at ambient temperature for 4 days. Hydrochloric acid (2.5%, 100 mL) was added and the resultant mixture extracted with diethyl ether (three 100 mL aliquots). The combined ether layers were evaporated and the extraction procedure repeated. The combined ether layers were dried (MgSO_4_), filtered and the solvent evaporated. The residue was rectified at reduced pressure affording *p*-methoxyphenyl methyl disulfide (0.88 g, 4.7 mmol, 66%, bp 112-118°C/2.5 Torr). The methoxyphenyl disulfide had ^1^H NMR (270 MHz) δ 2.43 (3H, s), 3.79 (3H, s), 6.87 (2H, d), 7.49 (2H, d); ^13^C NMR δ 22.9, 55.4, 114.8, 127.9, 132.4, 159.8; GCMS 186 (M^+.^, 91%), 139 (100%).

*Preparation of F1, F4, F5, F7, H2, H3, H4, H5 and H8*

These disulfides were prepared by reaction of the appropriate mercaptan, symmetrical disulfide (diphenyl or dimethyl) or unsymmetrical methyl disulfide with sulfuryl chloride. The sulfenyl chloride, so produced, was reacted with **1** or **2** to produce the target F relative or with methyl thioglycollate to produce the target H relative. The preparation of β-mesylethyl *p*-nitrophenyl disulfide F5, detailed below, serves to exemplify the procedure employed. Thereafter, the disulfides listed in the title are described in terms of their physical and spectroscopic properties. *p*-Nitrophenyl methyl disulfide (0.5 g, 2.5 mmol) was dissolved in dry methylene chloride (25 mL) and distilled sulfuryl chloride (0.43 g, 3.2 mmol) was added in small portions. The reaction mixture was refluxed for 30 min. The solvent was evaporated and a solution of mercaptosulfone **2** (0.35 g, 2.5 mmol) in dry pyridine (25 mL) was added. The reaction mixture was stirred at ambient temperature for 1 hour. Chloroform (100 mL) was added and the resultant mixture extracted with 2.5% hydrochloric acid (six 50 mL portions). The organic layer was dried (MgSO_4_), filtered and the solvent evaporated. Crude product was chromatographed on silica gel (100 g) employing chloroform (100 mL fractions) for elution. Fractions 10-13 were combined and concentrated affording β-mesylethyl *p*-nitrophenyl disulfide F5 (0.24 g, 0.8 mmol, 33%). Recrystallized F5 (14 mL, methanol) had mp 140-142°C; IR 1515, 1340, 1110 cm^-1^; ^1^H NMR (270 MHz) δ 2.96 (3H, s), 3.17 (2H, m), 3.37 (2H, m), 7.67 (2H, d), 8.21 (2H, d); ^13^C NMR δ 30.0, 41.5, 53.9, 124.4, 126.5, 144.8, 146.8; GCMS 293 (M^+.^, 55%), 213 (100%).

β-Mesylethyl carbomethoxymethyl disulfide F1 (mp 62-63.5°C, 11% yield) had IR 1727, 1305, 1132 cm^-1^; ^1^H NMR (270 MHz) δ 3.00 (3H, s), 3.15 (2H, m), 3.43 (2H, m), 3.53 (2H, s), 3.78 (3H, s); ^13^C NMR δ 29.7, 41.3, 41.6, 52.9, 54.1, 170.0; GCMS 244 (M^+.^, 22%), 212 (23%), 164 (100%), 79 (82%), 59 (64%).

β-Mesylethyl *p*-methoxyphenyl disulfide F4 (mp 88-90°C, 19% yield) had IR 1310, 1133 cm^-1^; ^1^H NMR (270 MHz) δ 2.91 (3H, s), 3.06 (2H, m), 3.38 (2H, m), 3.79 (3H, s), 6.87 (2H, d), 7.48 (2H, d); ^13^C NMR 29.7, 41.5, 53.9, 55.5, 115.0, 126.5, 133.1, 160.4; GCMS 278 (M^+.^, 44%), 171 (50%), 139 (100%). β-Tosylethyl phenyl disulfide F7 (mp 82.5-84°C), 25% yield) had IR 1303, 1147 cm^-1^; ^1^H NMR (270 MHz) δ 2.44 (3H, s), 2.91 (2H, m), 3.43 (2H, m), 7.35 (7H, m), 7.73 (2H, d); ^13^C NMR δ 21.7, 30.2, 55.6, 127.8, 128.1, 128.8, 129.2, 130.1, 135.7, 135.9, 145.1; GCMS 324 (M^+.^, 19%), 168 (76%), 141 (80%), 91 (100%). Carbomethoxymethyl propyl disulfide H2 (bulb to bulb distillation at 1.3 Torr with bath temperature 170°C, 23% yield) had IR 1737 cm^-1^; ^1^H NMR (270 MHz) δ 1.03 (3H, t), 1.73 (2H, sex), 2.74 (2H, t), 3.48 (2H, s), 3.78 (3H, s); ^13^C NMR δ 13.1, 22.3, 40.7, 41.3, 52.5, 170.1; GCMS 180 (M^+.^, 74%), 138 (29%), 106 (100%). Carbomethoxymethyl *p*-methoxyphenyl disulfide H3 (bp 165-170°C/1.4 Torr, 25% yield) had IR 1730 cm^-1^; ^1^H NMR (270 MHz) δ 3.43 (2H, s), 3.62 (3H, s), 3.80 (3H, s), 6.87 (2H, d), 7.50 (2H, d); ^13^C NMR δ 40.3, 52.4, 55.3, 114.6, 126.8, 133.0, 160.0, 169.5; GCMS 244 (M^+.^, 100%), 171 (76%), 139 (90%).

Carbomethoxymethyl phenyl disulfide H4 (bulb to bulb distillation at 0.9 Torr with bath temperature 210°C, 19% yield) had IR 1735 cm^-1^; ^1^H NMR (270 MHz) δ 3.49 (2H, s), 3.57 (3H, s), 7.34 (3H, m), 7.56 (2H, d); ^13^C NMR δ 40.5, 52.3, 127.5, 128.6, 129.0, 136.0, 169.3; GCMS 214 (M^+.^, 100%), 141 (75%), 109 (40%).

Carbomethoxymethyl heptyl disulfide H5 (bp 126-130°C/1.7 Torr, 38% yield) had IR 1740 cm^-1^; ^1^H NMR (270 MHz) δ 0.89 (3H, t), 1.29 (8H, broad envelope), 1.68 (2H, quin), 2.75 (2H, t), 3.47 (2H, s), 3.76 (3H,s); ^13^C NMR δ 14.0, 22.5, 28.4, 28.8, 29.0, 31.6, 38.7, 41.3, 52.5, 170.1; GCMS 236 (M^+.^, 92%), 138 (100%), 106 (68%), 57 (95%).

Carbomethoxymethyl *p*-nitrophenyl disulfide H8 (bulb to bulb distillation at 1.6 Torr with bath temperature 260°C, 17% yield) had IR 1737, 1510, 1340 cm^-1^; ^1^H NMR (270 MHz) δ 3.55 (2H, s), 3.62 (3H, s), 7.68 (2H, d), 8.20 (2H, d); ^13^C NMR δ 40.6, 52.6, 124.1, 124.5, 126.4, 145.4, 168.8; GCMS 259 (M^+.^, 100%), 200 (22%), 140 (32%).

*Preparation of F2 and F8*

These symmetrical disulfides were prepared by reaction of the appropriate mercaptan with molecular iodine in pyridine. The preparation of F8 serves to illustrate the procedure employed.

A solution of the mercaptosulfone **1** (0.75 g, 3.5 mmol) in pyridine (30 mL) was cooled in an ice/water bath. Iodine (0.45 g, 1.8 mmol) was added in small portions. The reaction mixture was stirred at ambient temperature for 30 min. Chloroform (200 mL) was added and the resultant mixture extracted with 5% hydrochloric acid (two 100 mL aliquots). Saturated sodium thiosulfate (5 mL) was added to the organic layer which was dried (MgSO_4_), filtered and the solvent evaporated. Crude product was chromatographed on silica gel (75 g) employing 1:1 chloroform/petroleum ether (75 mL fractions) for elution. Fractions 13-16 were combined, concentrated and the product recrystallized (methanol) to give the symmetrical disulfone disulfide F8 (0.72 g, 1.6 mmol, 95%, mp 120.8-122.5°C) which had IR 1322, 1145 cm^-1^; ^1^H NMR (270 MHz) δ 2.45 (3H, s), 2.84 (2H, m), 3.36 (2H, m), 7.37 (2H, d), 7.76 (2H, d); GCMS (CI) 431 (M^+.^+ H, 100%), 275 (21%).

Di-(β-mesylethyl) disulfide F2 (mp 147-150°C, 40% yield) had IR 1297, 1133 cm^-1^; ^1^H

NMR (270 MHz, DMSO-d_6_) 3.05 (3H, s), 3.12 (2H, m), 3.45 (2H, m); ^13^C NMR δ 29.7, 39.5, 53.1.

*Preparation of F3*

Sodium metal (0.16 g, 7.1 mmol) was dissolved in methanol (10 mL) and the solvent evaporated. DMSO (5 mL) was added and the mixture vigorously stirred with a mechanical stirrer for 6 hours. β-Mesylethyl mercaptan **2** (1.00 g, 7.1 mmol) and diphenyl disulfide (1.56 g, 7.1 mmol) were added. The reaction mixture was stoppered and gently stirred at ambient temperature for 8 days. Hydrochloric acid (2%, 100 mL) was added and the mixture extracted with diethyl ether (three 100 mL aliquots). The combined organic extracts were washed with 2.5% W/V sodium hydroxide (three 50 mL portions), dried (MgSO_4_), filtered and the solvent evaporated. Crude product was chromatographed on silica gel (200 g) employing petroleum ether (100 mL fractions) for elution. Fractions 44-50 were combined and concentrated affording β-mesylethyl phenyl sulfide F3 (0.81 g, 3.3 mmol, 46%). Recrystallized (methanol) F3 had mp 46-49°C; IR 1313, 1139 cm^-1^; ^1^H NMR (270 MHz) δ 2.93 (3H, s), 3.26 (2H, m), 3.35 (2H, m), 7.34 (5H, m); ^13^C NMR δ 26.3, 41.2, 54.2, 127.4, 129.4, 130.5, 133.3; GCMS 216 (M^+.^, 16%), 136 (100%), 109 (94%).

*Preparation of F6*

Sodium metal (22 mg, 0.95 mmol) was dissolved in methanol (10 mL). Methyl mercaptan (20 mL) was bubbled slowly into the solution. The solvent was evaporated and the residue dried *in vacuo*. Residual sodium methanethiolate was dissolved in DMSO (10 mL). Ditosylethyl disulfide F8 (0.25 g, 0.58 mmol) was added to dimethyl disulfide (5 mL) and a portion of the methanethiolate solution (0.4 mL) was added. The reaction mixture was stirred at ambient temperature for 1 week. Hydrochloric acid (2.5%, 250 mL) was added and the resultant mixture extracted with diethyl ether (three 100 mL aliquots). The combined organic layers were dried (MgSO_4_), filtered and the solvent evaporated affording crude disulfide. The product was recrystallized from methanol (0.5 mL) affording sulfone disulfide F6 (0.10 g, 0.37 mmol, 32%, mp 42.3-44.6°C) which had IR 1301, 1147 cm^-1^; ^1^H NMR (270 MHz) δ 2.33 (3H, s), 2.45 (3H, s), 2.90 (2H, m), 3.44 (2H, m), 7.36 (2H, d), 7.78 (2H, d); ^13^C NMR δ 21.8, 23.0, 29.2, 56.2, 128.4, 130.1, 136.0, 145.1; GCMS 262 (M^+.^, 15%), 155 (16%), 106 (100%), 91 (74%).

*Preparation of H6 and H7*

These ester disulfides were prepared by reaction of the corresponding methyl esters with 1-hexanol in the presence of sulfuric acid. The preparation of H7 serves to illustrate the procedure. Carbomeythoxymethyl phenyl disulfide H4 (1.01 g, 4.73 mmol) was dissolved in 1-hexanol (75 mL) and concentrated sulfuric acid (0.5 mL) was added. The reaction mixture was refluxed for 24 h. Chloroform (150 mL) was added and the resultant mixture extracted with 2.5% W/V sodium hydroxide (three - 100 mL aliquots). The organic layer was dried (MgSO_4_), filtered and the chloroform evaporated. Residual hexanol was removed with a bath (82°C) at reduced pressure (40 Torr). Crude ester disulfide was chromatographed on silica gel (100 g) employing 1:1 chloroform/petroleum ether (100 mL fractions) for elution. Fractions 3 and 4 were combined and concentrated. The residue was rectified at reduced pressure affording clean hexyl ester H7 (0.95 g, 3.33 mmol, 70%, bp 188-190°C/3.6 Torr) which had IR 1731 cm^-1^; ^1^H NMR (270 MHz) δ 0.89 (3H, t), 1.28 (6H, broad envelope), 1.57 (2H, quin), 3.49 (2H, s), 3.98 (2H, t), 7.27 (3H, m), 7.53 (2H, d); GCMS 284 (M^+.^, 100%), 200 (57%), 141 (63%). Hexyl 3,4-dithiapentanoate H6 (chromatographed, distilled, bp 146-150°C/1.6 Torr, 33%) had IR 1730 cm^-1^; ^1^H NMR (270 MHz) δ 0.87 (3H, t), 1.00 (3H, t), 1.33 (6H, broad envelope), 1.68 (2H, sex), 1.73 (2H, quin), 2.74 (2H, t), 3.46 (2H, s), 4.14 (2H, t); ^13^C NMR δ 13.0, 14.0, 22.3, 22.5, 25.5, 28.5, 31.4, 40.7, 41.6, 65.7, 169.8; GCMS (250 (M^+.^, 100%), 124 (53%), 43 (85%).

*Preparation of N1 and N2*

The preparation and properties of these molecules have been described earlier (3).

## References

1. Wong, W. W., Macdonald, S., Langler, R. F., and Penn, L. Z. Novel synthetic organosulfur compounds induce apoptosis of human leukemic cells. Anticancer Res, *20:* 1367-1374, 2000.

2. Langler, R. F. a-Thiodisulfides: construction and biological activities. Journal of Sulfur Chemistry, *29:* 99 - 127, 2008.

3. Bewick, S. A., Duffy, S., Fletcher, S. P., Langler, R. F., Morrison, H. G., Oâ€™Brien, E. M., Ross, C., and Stephenson, V. C. The S2 Oxygen Atoms Are Essential for the Pronounced Fungitoxicity of the Sulfur-Rich Natural Product, Dysoxysulfone. Australian Journal of Chemistry, *58:* 218-223, 2005.
