# Supplementary material for: Characterization of the apoptotic response of human leukemia cells to organosulfur compounds
Source: BMC Cancer. 2010 Jul 2;10:351. doi: 10.1186/1471-2407-10-351 (PMC2928001; doi:10.1186/1471-2407-10-351)
Supplement: Additional file 2 — Figures S1-S9. Microsoft PowerPoint file containing Supplementary Figures 1-9, along with FigureLegends. [file 1471-2407-10-351-S2.DOC]

Supplementary Table 1. Organosulfur compounds assayed for anti-cancer activity.^1^

| Compound Name | Structure | Molecular Weight (g/mol) |
| --- | --- | --- |
| F | CH_3_SO_2_CH_2_CH_2_SSCH_3_ | 186.3 |
| H | CH_3_OC(O)CH_2_SSCH_3_ | 152.8 |
| N | CH_3_SCH_2_SSCH_3_ | 140.3 |
| N1 | CH_3_SCH_2_SSCH_2_SCH_2_SO_2_CH_3_ | 264.5 |
| N2 | CH_3_SCH_2_SSCH_2_CH_2_CH_2_SO_2_CH_3_ | 246.4 |
| F1 | CH_3_SO_2_CH_2_CH_2_SSCH_2_CO_2_CH_3_ | 244.4 |
| F2 | CH_3_SO_2_CH_2_CH_2_SSCH_2_CH_2_SO_2_CH_3_ | 278.4 |
| F3 | CH_3_SO_2_CH_2_CH_2_SSPh | 248.4 |
| F4 | CH_3_SO_2_CH_2_CH_2_SS(C_6_H_4_)OCH_3_ | 278.4 |
| F5 | CH_3_SO_2_CH_2_CH_2_SS(C_6_H_4_)NO_2_ | 293.4 |
| F6 | CH_3_(C_6_H_4_)SO_2_CH_2_CH_2_SSCH_3_ | 262.4 |
| F7 | CH_3_(C_6_H_4_)SO_2_CH_2_CH_2_SSPh | 324.5 |
| F8 | CH_3_(C_6_H_4_)SO_2_CH_2_CH_2_SSCH_2_CH_2_SO_2_(C_6_H_4_)CH_3_ | 430.6 |
| H1 | CH_3_OC(O)CH_2_CH_2_SSCH_3_ | 166.3 |
| H2 | CH_3_OC(O)CH_2_SSCH_2_CH_2_CH_3_ | 180.3 |
| H3 | CH_3_OC(O)CH_2_SS(C_6_H_4_)OCH_3_ | 246.3 |
| H4 | CH_3_OC(O)CH_2_SSPh | 214.3 |
| H5 | CH_3_OC(O)CH_2_SS(CH_2_)_6_CH_3_ | 236.4 |
| H6 | CH_3_(CH_2_)_5_OC(O)CH_2_SSCH_2_CH_2_CH_3_ | 250.4 |
| H7 | CH_3_(CH_2_)_5_OC(O)CH_2_SSPh | 284.4 |
| H8 | CH­_3_OC(O)CH_2_SS(C_6_H_4_)NO_2_ | 259.3 |

^1^ For previous results of Compounds A-Q, including F, H and N, see Wong *et al.,* Anticancer Research 2000 **20**(3A): 1367-74 and Griffiths *et al*., Aust. J. Chem 2005 **58**:128-136
