# Supplementary material for: Characterization of the apoptotic response of human leukemia cells to organosulfur compounds
Source: BMC Cancer. 2010 Jul 2;10:351. doi: 10.1186/1471-2407-10-351 (PMC2928001; doi:10.1186/1471-2407-10-351)
Supplement: Additional file 3 — Table S1. List of all organosulfur compounds discussed in the manuscript. [file 1471-2407-10-351-S3.PPT]

## Slide 1
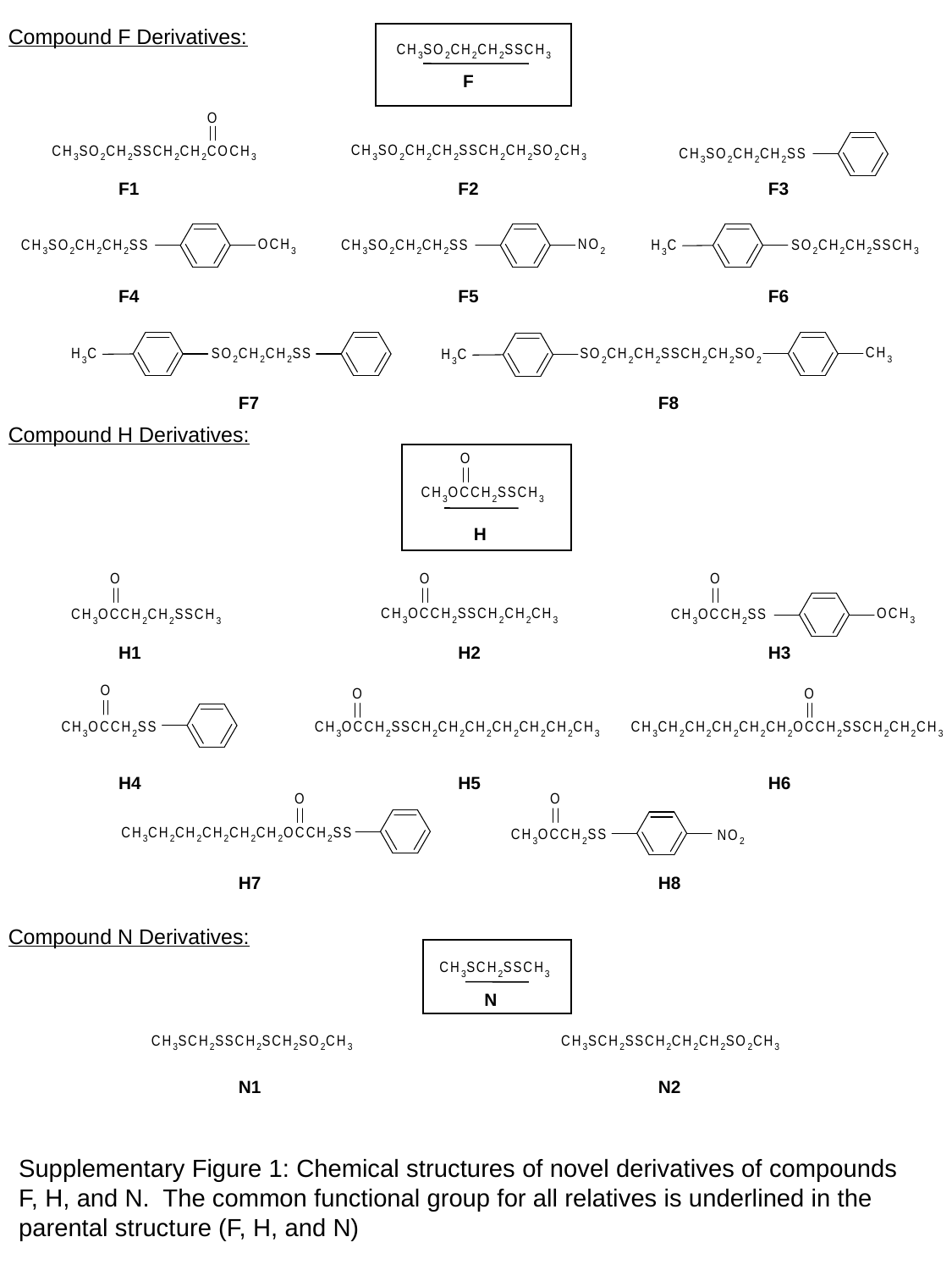

Compound F Derivatives:
F
F1
F2
F3
F4
F5
F6
F7
F8
Compound H Derivatives:
H
H1
H2
H3
H4
H5
H6
H7
H8
Compound N Derivatives:
N
N1
N2
Supplementary Figure 1: Chemical structures of novel derivatives of compounds F, H, and N. The common functional group for all relatives is underlined in the parental structure (F, H, and N)

## Slide 2
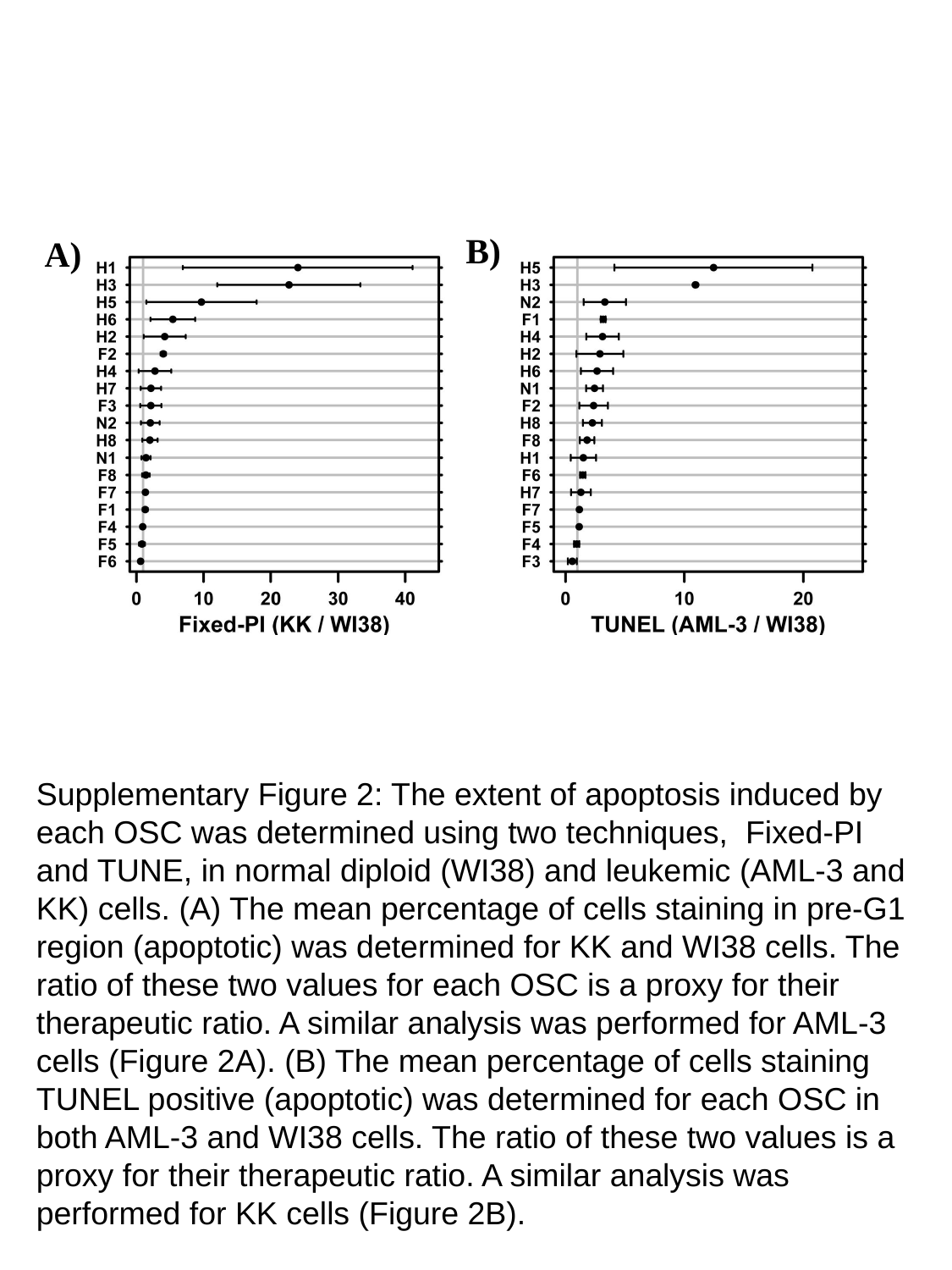

B)
A)
Supplementary Figure 2: The extent of apoptosis induced by each OSC was determined using two techniques, Fixed-PI and TUNE, in normal diploid (WI38) and leukemic (AML-3 and KK) cells. (A) The mean percentage of cells staining in pre-G1 region (apoptotic) was determined for KK and WI38 cells. The ratio of these two values for each OSC is a proxy for their therapeutic ratio. A similar analysis was performed for AML-3 cells (Figure 2A). (B) The mean percentage of cells staining TUNEL positive (apoptotic) was determined for each OSC in both AML-3 and WI38 cells. The ratio of these two values is a proxy for their therapeutic ratio. A similar analysis was performed for KK cells (Figure 2B).

## Slide 3
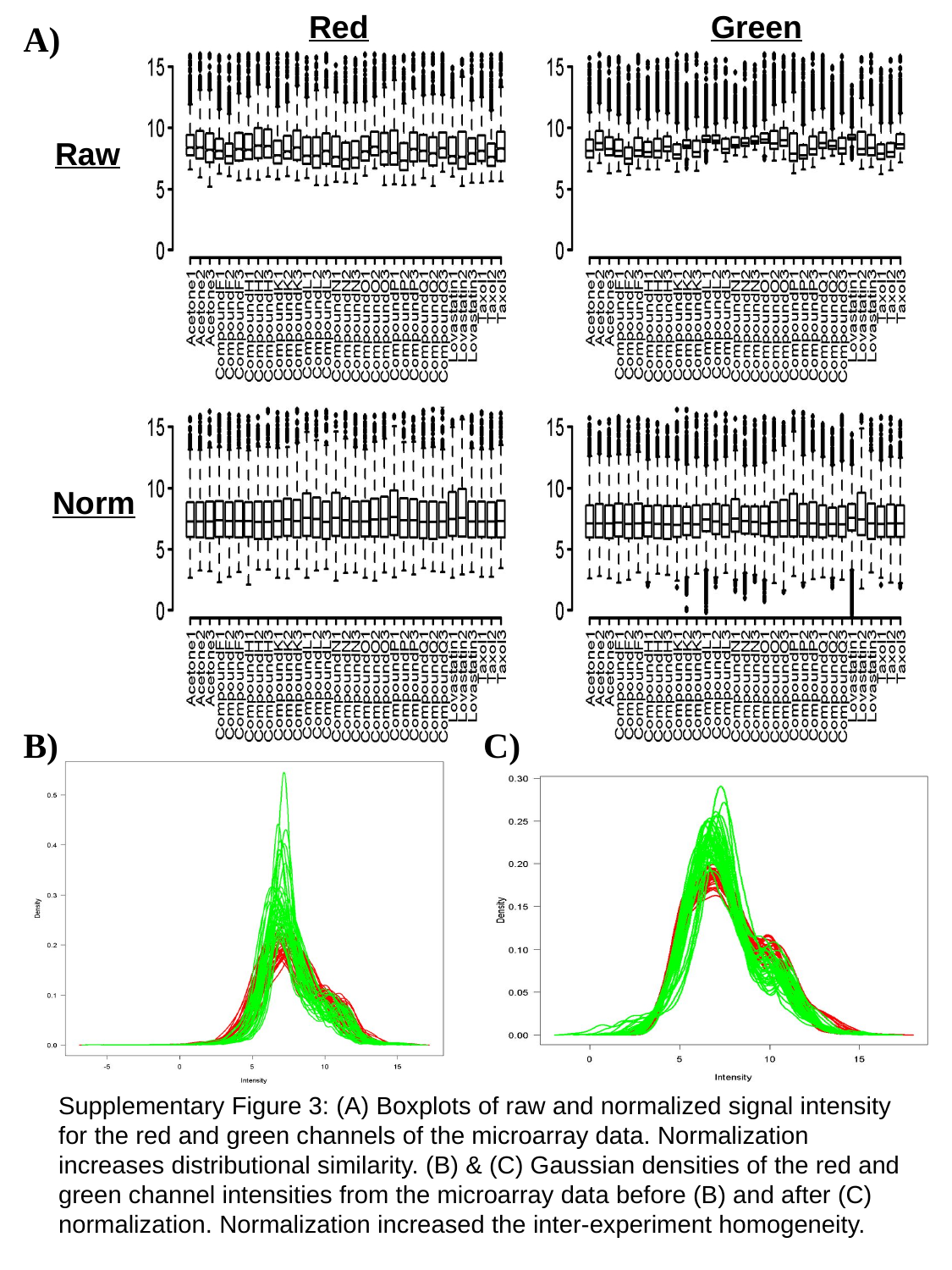

Red
Green
A)
Raw
Norm
B)
C)
Supplementary Figure 3: (A) Boxplots of raw and normalized signal intensity for the red and green channels of the microarray data. Normalization increases distributional similarity. (B) & (C) Gaussian densities of the red and green channel intensities from the microarray data before (B) and after (C) normalization. Normalization increased the inter-experiment homogeneity.

## Slide 4
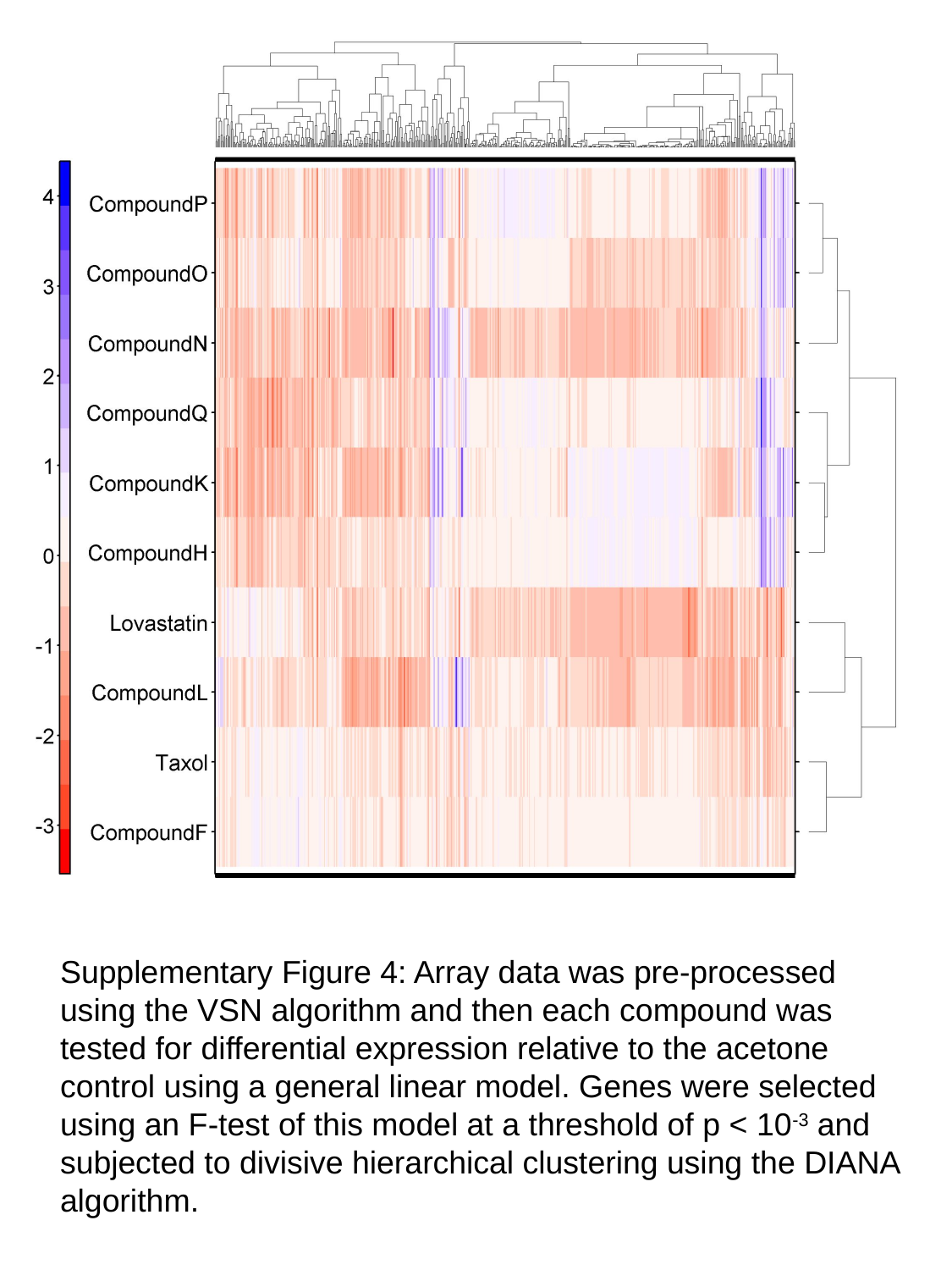

Supplementary Figure 4: Array data was pre-processed using the VSN algorithm and then each compound was tested for differential expression relative to the acetone control using a general linear model. Genes were selected using an F-test of this model at a threshold of p < 10-3 and subjected to divisive hierarchical clustering using the DIANA algorithm.

## Slide 5
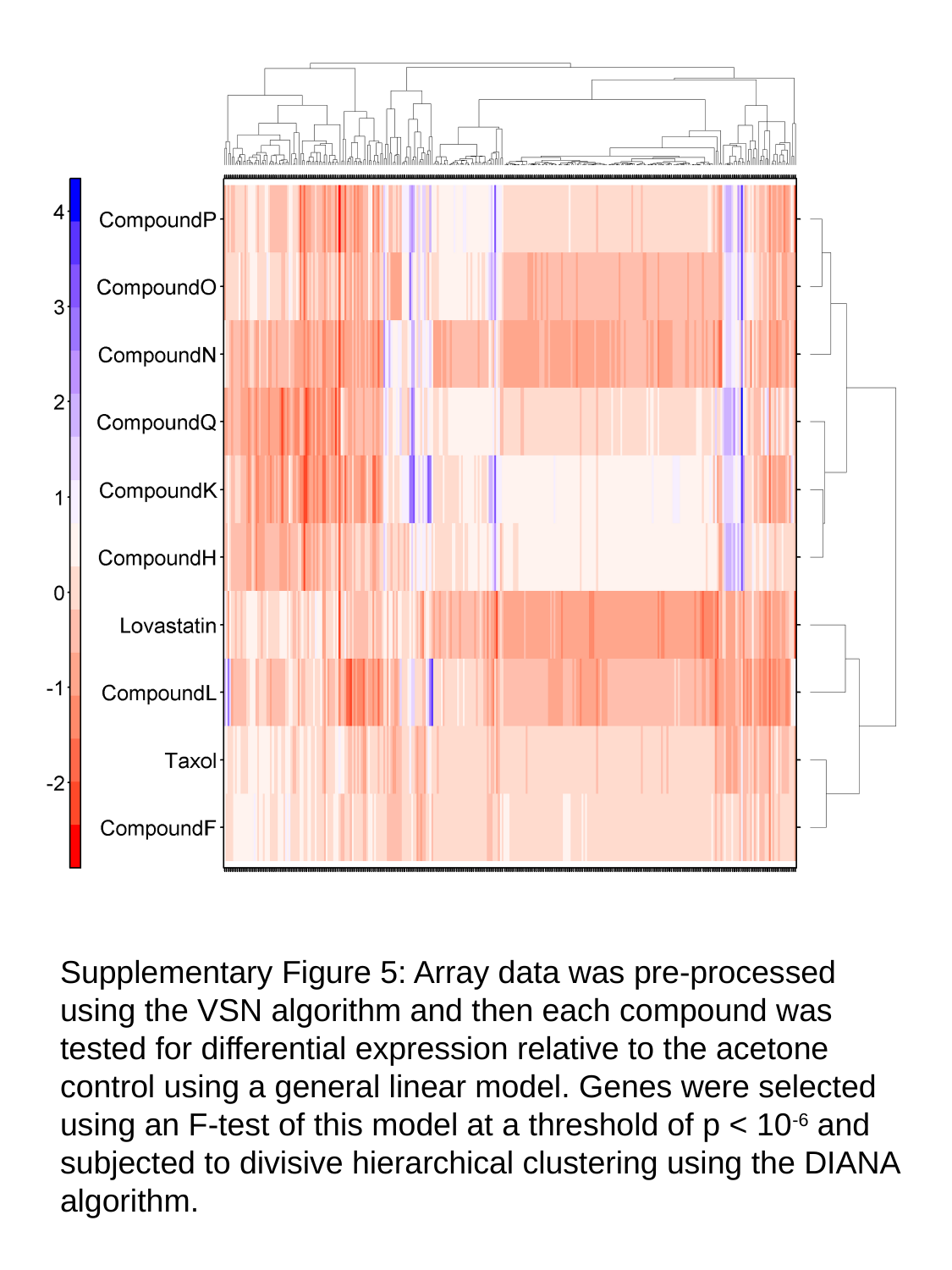

Supplementary Figure 5: Array data was pre-processed using the VSN algorithm and then each compound was tested for differential expression relative to the acetone control using a general linear model. Genes were selected using an F-test of this model at a threshold of p < 10-6 and subjected to divisive hierarchical clustering using the DIANA algorithm.

## Slide 6
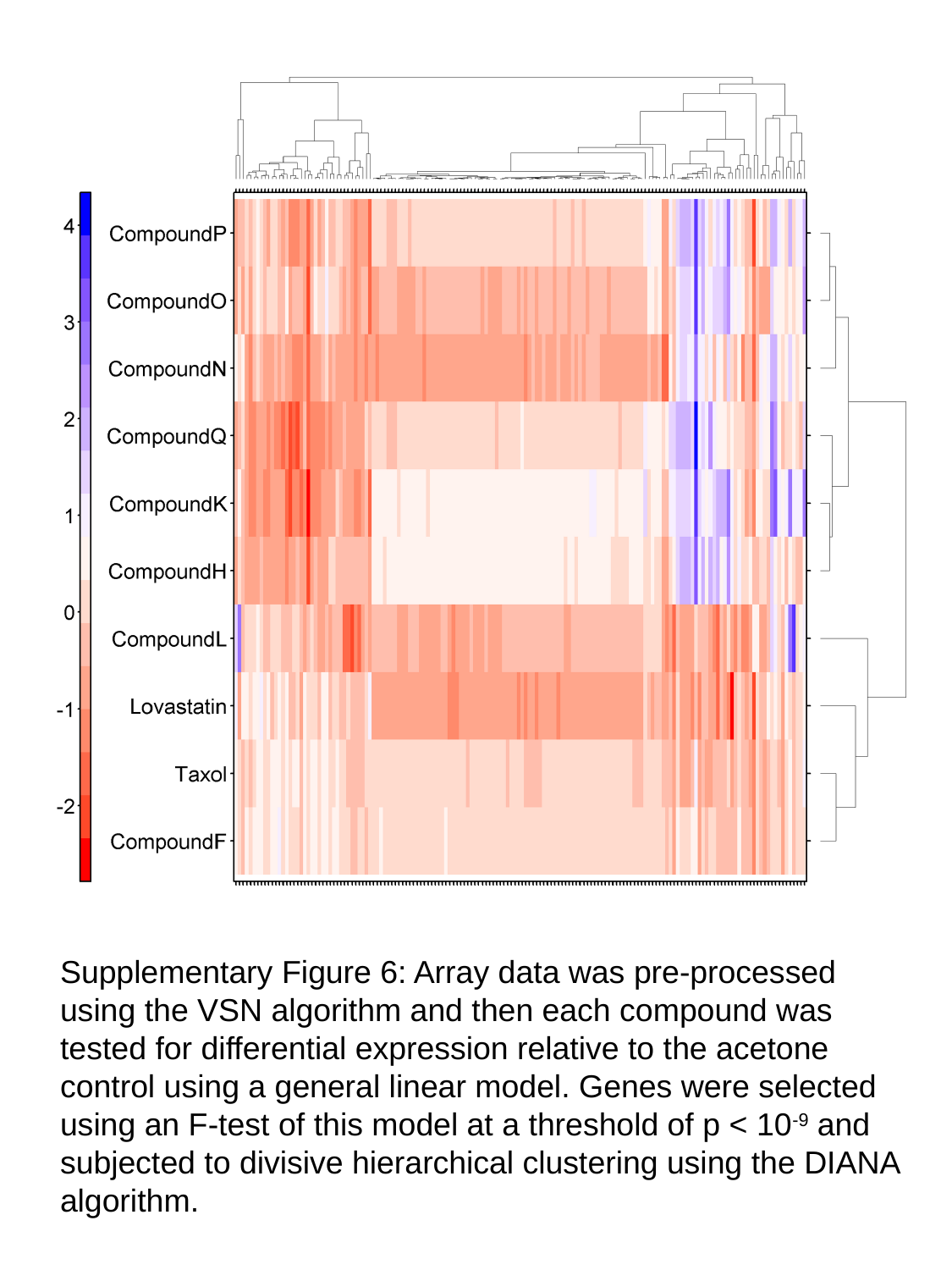

Supplementary Figure 6: Array data was pre-processed using the VSN algorithm and then each compound was tested for differential expression relative to the acetone control using a general linear model. Genes were selected using an F-test of this model at a threshold of p < 10-9 and subjected to divisive hierarchical clustering using the DIANA algorithm.

## Slide 7
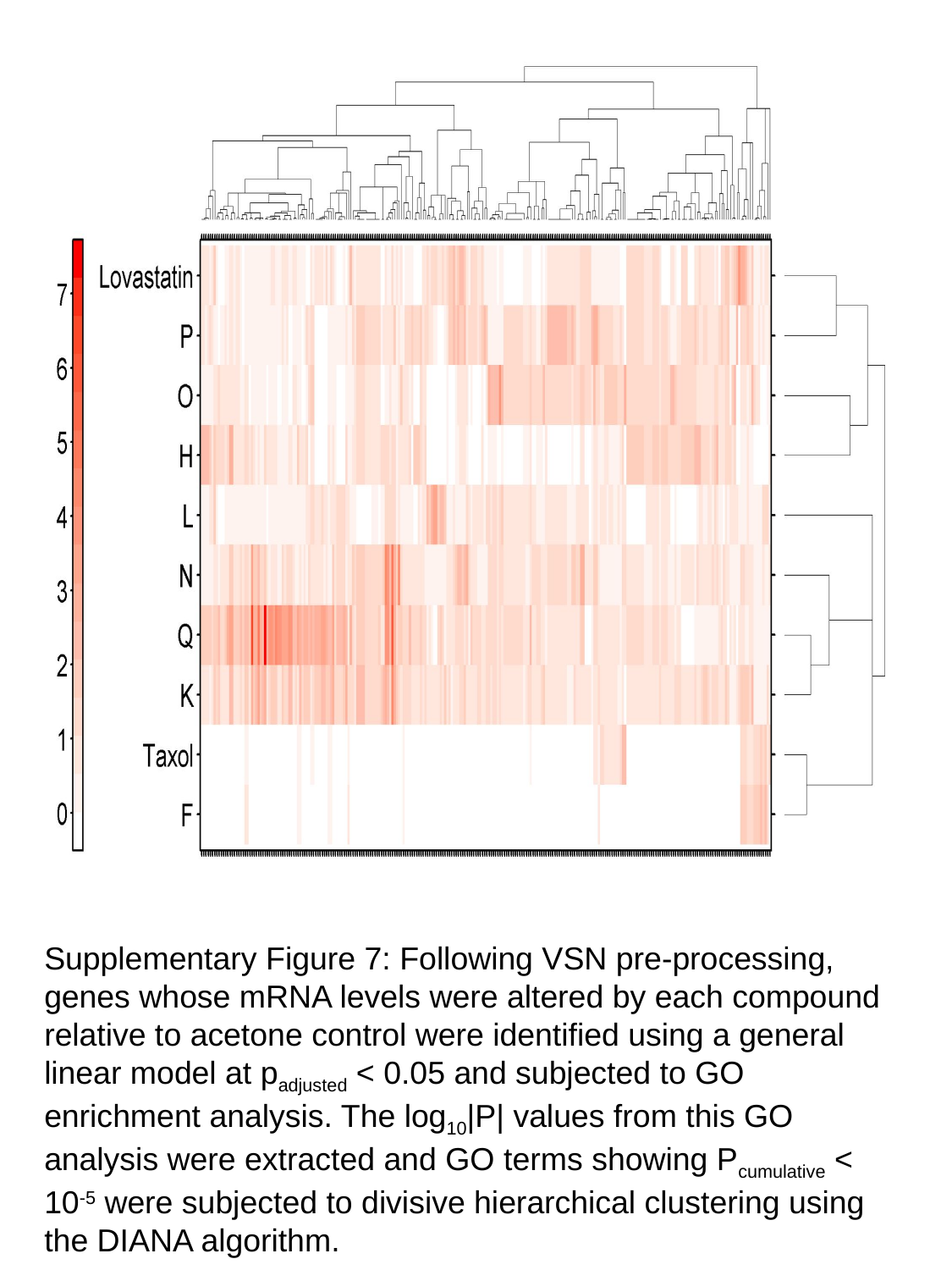

Supplementary Figure 7: Following VSN pre-processing, genes whose mRNA levels were altered by each compound relative to acetone control were identified using a general linear model at padjusted < 0.05 and subjected to GO enrichment analysis. The log10|P| values from this GO analysis were extracted and GO terms showing Pcumulative < 10-5 were subjected to divisive hierarchical clustering using the DIANA algorithm.

## Slide 8
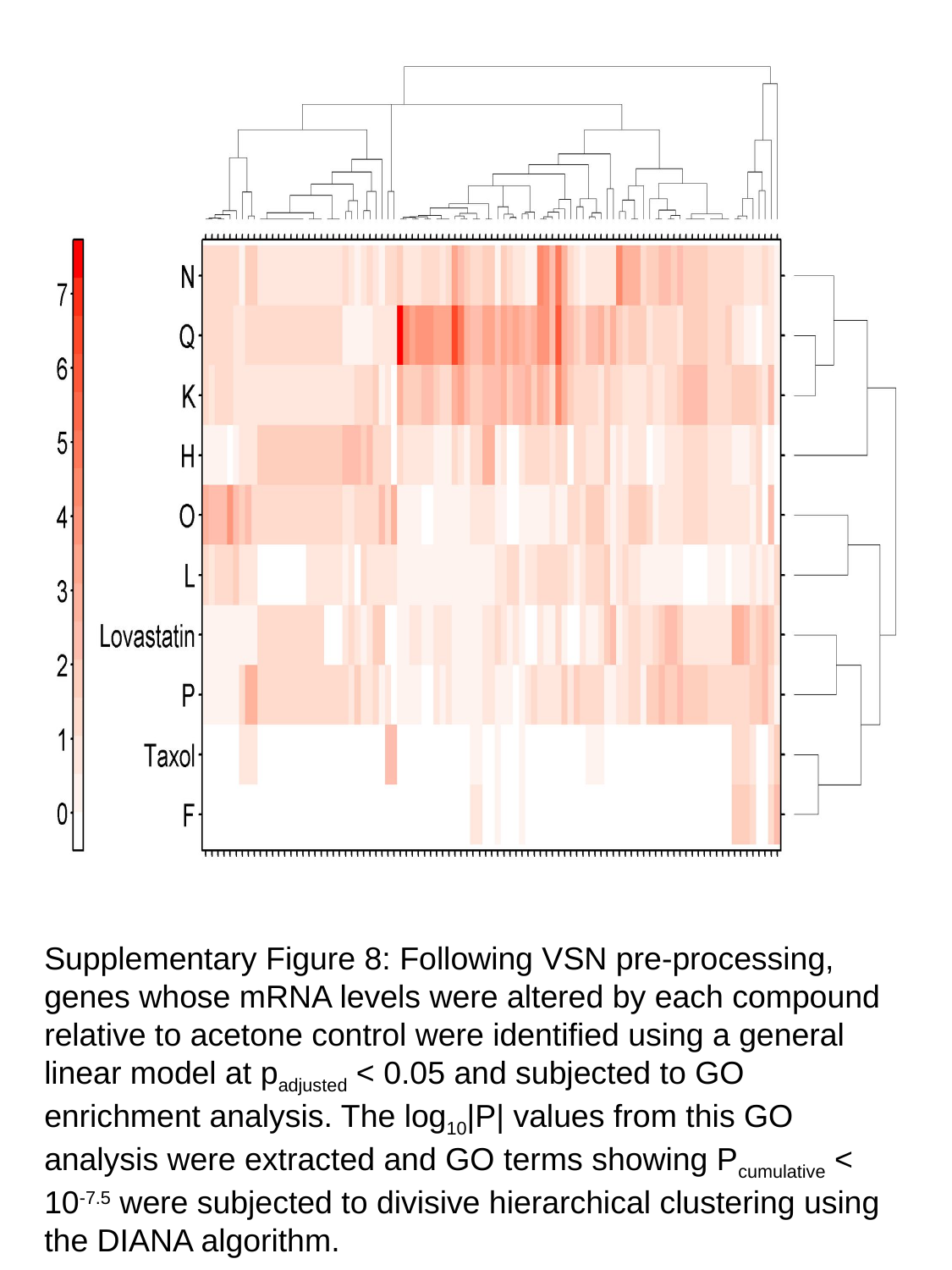

Supplementary Figure 8: Following VSN pre-processing, genes whose mRNA levels were altered by each compound relative to acetone control were identified using a general linear model at padjusted < 0.05 and subjected to GO enrichment analysis. The log10|P| values from this GO analysis were extracted and GO terms showing Pcumulative < 10-7.5 were subjected to divisive hierarchical clustering using the DIANA algorithm.

## Slide 9
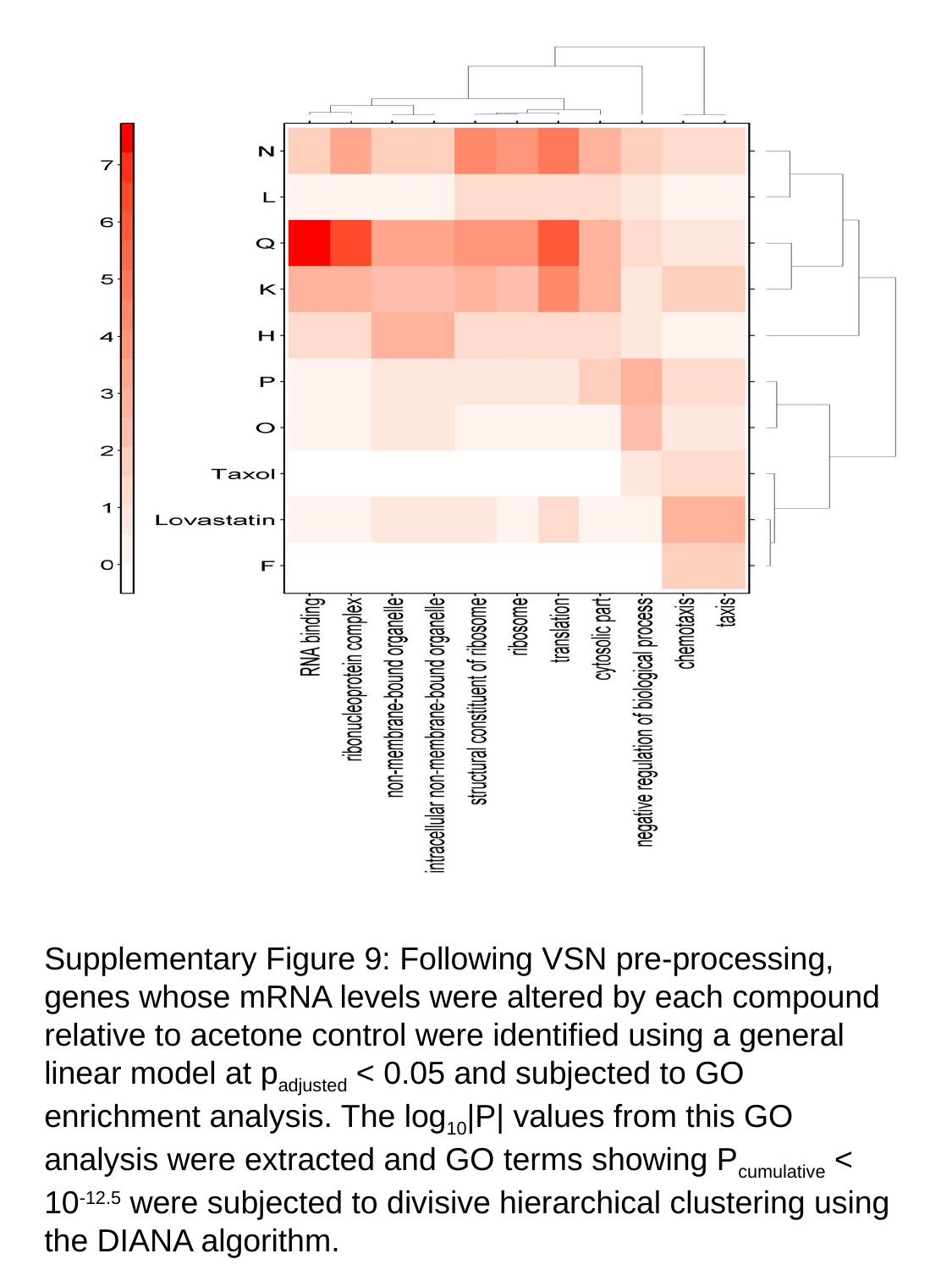

Supplementary Figure 9: Following VSN pre-processing, genes whose mRNA levels were altered by each compound relative to acetone control were identified using a general linear model at padjusted < 0.05 and subjected to GO enrichment analysis. The log10|P| values from this GO analysis were extracted and GO terms showing Pcumulative < 10-12.5 were subjected to divisive hierarchical clustering using the DIANA algorithm.
