# Supplementary material for: Characterization of the apoptotic response of human leukemia cells to organosulfur compounds
Source: BMC Cancer. 2010 Jul 2;10:351. doi: 10.1186/1471-2407-10-351 (PMC2928001; doi:10.1186/1471-2407-10-351)
Supplement: Additional file 4 — Table S2. Complete MTT50 data. [file 1471-2407-10-351-S4.DOC]

**Supplementary Table 2**: The dose (μM) required to reduce the MTT activity to 50% (MTT_50_) of the non-transformed diploid cell line (WI38) as well as the acute myelogenous leukemia derived cell line (AML-3). The MTT_50_ value was determined by exposing the cells to a wide concentration range of OSCs (0-200 μM) for 48 h and then conducting MTT assays. Results shown are the mean MTT_50_ for three independent experiments performed with three replicates in each experiment. ND, not determined

| **Compound** | **WI38 (µM)** | **AML-3 (µM)** | **WI38 / AML-3** |
| --- | --- | --- | --- |
| N1 | 42.5 ± 8.0 | 16.5 ± 2.4 | 2.58 |
| N2 | >200 | 53.4 ± 3.7 | >3.74 |
| F1 | 173.7 ± 11.0 | 45.9 ± 2.0 | 3.78 |
| F2 | >200 | >200 | ND |
| F3 | >200 | >200 | ND |
| F4 | 34.4 ± 3.8 | 8.6 ± 1.1 | 4.0 |
| F5 | 17.4 ± 1.2 | 8.9 ± 0.9 | 1.96 |
| F6 | 35.6 ± 3.6 | 8.0 ± 1.0 | 4.45 |
| F7 | 42.5 ± 56.0 | 14.5 ± 1.6 | 2.93 |
| F8 | 6.5 ± 0.9 | 27.2 ± 4.4 | 0.24 |
| H1 | >200 | >200 | ND |
| H2 | >200 | 70.1 ± 7.2 | >2.85 |
| H3 | 58.5 ± 4.2 | 24.2 ± 1.7 | 2.42 |
| H4 | 83.7 ± 5.2 | 38.0 ± 2.8 | 2.20 |
| H5 | > 200 | 122.2 ± 7.1 | >1.64 |
| H6 | > 200 | 64.1 ± 4.7 | >3.12 |
| H7 | 102.9 ± 5.1 | 43.8 ± 2.5 | 2.35 |
| H8 | 21.7 ± 4.2 | 29.9 ± 5.6 | 0.72 |
