# Supplementary material for: Characterization of the apoptotic response of human leukemia cells to organosulfur compounds
Source: BMC Cancer. 2010 Jul 2;10:351. doi: 10.1186/1471-2407-10-351 (PMC2928001; doi:10.1186/1471-2407-10-351)
Supplement: Additional file 5 — Table S3. Complete Fixed-PI and TUNEL data. [file 1471-2407-10-351-S5.DOC]

**Supplementary Table 3:** Apoptosis was assessed by fixed-PI (percentage of pre-G1 cells) and TUNEL (percentage of TUNEL positive cells). AML-3 and KK are leukemic cell lines; WI38 cells are normal diploid fibroblasts. Samples were exposed to OSCs for 48 hours. All experiments were independently repeated two or more times with the exceptions of compound F7 at 60 μM (fixed-PI) and compound F1 at 150μM and F5, F7, and H3 at 75 μM (TUNEL) which were assayed once. ND, not determined. * A concentration of 75 µM was used for TUNEL for H7.

| **Compound** | **Dose (µM)** | **Fixed PI** | | | **TUNEL** | | |
| --- | --- | --- | --- | --- | --- | --- | --- |
|  |  | **AML-3** | **KK** | **WI38** | **AML-3** | **KK** | **WI38** |
| N1 | 50 | 22.1 ± 6.4 | 17.8 ± 1.8 | 6.3 ± 2.7 | 56.1 ± 16.5 | 36.5 ± 6.1 | 6.3 ± 2.7 |
|  | 100 | 40.2 ± 8.5 | 45.1 ± 16.9 | 31.6 ± 9.2 | 77.1 ± 0.6 | 50.8 ± 5.2 | 31.6 ± 9.2 |
| N2 | 100 | 13.7 ± 1.3 | 14.3 ± 5.4 | 5.6 ± 2.0 | 16.3 ± 2.9 | 20.6 ± 0.7 | 5.6 ± 2.0 |
|  | 150 | 27.1 ± 12.6 | 15.5 ± 6.4 | 7.5 ± 4.0 | 24.8 ± 2.0 | 41.9 ± 2.2 | 7.5 ± 4.0 |
| F1 | 100 | 6.9 ± 1.5 | 14.4 ± 6.3 | 4.3 ± 0.6 | 14.2 ± 3.2 | 12.0 ± 4.0 | 4.3 ± 0.6 |
|  | 150 | 30.3 ± 4.3 | 16.2 ± 4.6 | 12.4 ± 0.8 | 39.3 ± 0.0 | 19.9 | 12.4 ± 0.8 |
| F2 | 100 | 2.5 ± 0.9 | 2.4 ± 0.2 | 0.5 ± 0.1 | 1.3 ± 0.6 | 1.3 ± 0.2 | 0.5 ± 0.1 |
|  | 200 | 4.0 ± 2.0 | 4.4 ± 0.2 | 1.1 ± 0.1 | 2.6 ± 1.3 | 2.4 ± 0.7 | 1.1 ± 0.1 |
| F3 | 100 | 1.7 ± 1.0 | 2.1 ± 0.4 | 0.7 ± 0.1 | 1.0 ± 0.4 | 1 ± 0.4 | 0.7 ± 0.1 |
|  | 200 | 1.3 ± 0.1 | 1.5 ± 0.9 | 0.7 ± 0.3 | 0.4 ± 0.2 | 1.5 ± 0.5 | 0.7 ± 0.3 |
| F4 | 20 | 6.6 ± 0.5 | 4.7 ± 1.1 | 0.8 ± 0.3 | 2.0 ± 0.7 | 7.8 ± 3.0 | 0.8 ± 0.3 |
|  | 40 | 34.9 ± 12.4 | 33.2 ± 6.7 | 35.4 ± 6.6 | 33.1 ± 4.7 | 30.6 ± 1.8 | 35.4 ± 6.6 |
| F5 | 10 | 0.8 ± 0.2 | 5.2 ± 3.2 | 1.1 ± 0.6 | 0.2 | 0.9 | 1.1 ± 0.6 |
|  | 40 | 35.8 ± 1.5 | 44.6 ± 23.2 | 54.5 ± 6.8 | 62.6 | ND | 54.5 ± 6.8 |
| F6 | 40 | 20.6 ± 2.7 | 12.9 ± 0.4 | 10.7 ± 1.6 | 33.1 ± 5.4 | 31.1 ± 0.6 | 10.7 ± 1.6 |
|  | 80 | 35.7 ± 5.0 | 19.5 ± 6.9 | 31.3 ± 3.5 | 45.4 ± 5.0 | 28.6 ± 1.1 | 31.3 ± 3.5 |
| F7 | 30 | 6.9 ± 2.8 | 11 ± 2.6 | 7.1 ± 2.0 | 7.9 | 17.7 | 7.1 ± 2.0 |
|  | 60 | 53.6 | 51.1 ± 4.1 | 38.2 ± 3.6 | 44.3 | 62.3 | 38.2 ± 3.6 |
| F8 | 20 | 12.6 ± 1.6 | 8.2 ± 2.3 | 15.3 ± 2.5 | 11.9 ± 0.5 | 10.5 ± 0.5 | 15.3 ± 2.5 |
|  | 40 | 34.6 ± 10.6 | 34.4 ± 7.8 | 24.5 ± 8.1 | 44.3 ± 3.3 | 38.7 ± 1.6 | 24.5 ± 8.1 |
| H1 | 100 | 1.8 ± 0.9 | 1.7 ± 1.1 | 0.5 ± 0.1 | 0.8 ± 0.4 | 1.2 ± 0.7 | 0.5 ± 0.1 |
|  | 200 | 1.9 ± 1.0 | 9.6 ± 6.4 | 0.4 ± 0.1 | 0.6 ± 0.4 | 3.6 ± 1.7 | 0.4 ± 0.1 |
| H2 | 50 | 5.4 ± 3.6 | 13.7 ± 8.6 | l.0 ± 0.6 | 4.1 ± 1.1 | 9.8 ± 2.2 | l.0 ± 0.6 |
|  | 100 | 14.4 ± 6.2 | 19.8 ± 12.3 | 4.7 ± 1.9 | 13.6 ± 7.5 | 22.0 ± 1.2 | 4.7 ± 1.9 |
| H3 | 50 | 30.5 ± 15.4 | 26.0 ± 20.1 | 6.3 ± 3.7 | 19.7 ± 3.5 | 29.2 ± 4.4 | 6.3 ± 3.7 |
|  | 75 | 64.1 ± 12.6 | 63.5 ± 12.1 | 2.8 ± 1.2 | 30.6 | 39.3 ± 5.6 | 2.8 ± 1.2 |
| H4 | 50 | 16.8 ± 8.6 | 25.1 ± 12.9 | 2.8 ± 1.2 | 7.0 ± 2.2 | 13.9 ± 4.8 | 2.8 ± 1.2 |
|  | 75 | 38.8 ± 16.5 | 28.7 ± 22 | 10.4 ± 4.5 | 32.4 ± 2.4 | 42.3 ± 7.9 | 10.4 ± 4.5 |
| H5 | 100 | 11.0 ± 9.1 | 11.7 ± 8.6 | 0.8 ± 0.3 | 6.4 ± 2.4 | 8.0 ± 0.4 | 0.8 ± 0.3 |
|  | 200 | 42.5 ± 18.0 | 37.8 ± 19.8 | 3.9 ± 2.6 | 48.5 ± 2.8 | 25.7 ± 8.1 | 3.9 ± 2.6 |
| H6 | 50 | 5.4 ± 3.3 | 7.6 ± 3.6 | 0.5 ± 0.3 | 3.4 ± 1.4 | 13.2 ± 0.6 | 0.5 ± 0.3 |
|  | 100 | 18.1 ± 5.0 | 21.7 ± 10.1 | 4.0 ± 1.6 | 10.6 ± 3.4 | 23.2 ± 0.7 | 4.0 ± 1.6 |
| H7 | 50 | 25.6 ± 12.4 | 20.1 ± 12.9 | 2.0 ± 0.8 | 14.6 ± 5.4 | 11.8 ± 1.2 | 2.0 ± 0.8 |
|  | 100* | 68.5 ± 13.1 | 66.8 ± 19.7 | 31.0 ± 19.9 | 40.1 ± 0.7 | 66.8 ± 19.7 | 31.0 ± 19.9 |
| H8 | 20 | 6.6 ± 1.9 | 20.7 ± 7.5 | 18.4 ± 9.0 | 60.5 ± 4.2 | 22.1 ± 8.5 | 18.4 ± 9.0 |
|  | 40 | 37.6 ± 15.6 | 56.2 ± 25.5 | 28.0 ± 9.6 | 63.2 ± 5.5 | 33.5 ± 3.7 | 28.0 ± 9.6 |
